# Stable Gastric Pentadecapeptide BPC 157 and Striated, Smooth, and Heart Muscle

**DOI:** 10.3390/biomedicines10123221

**Published:** 2022-12-12

**Authors:** Mario Staresinic, Mladen Japjec, Hrvoje Vranes, Andreja Prtoric, Helena Zizek, Ivan Krezic, Slaven Gojkovic, Ivan Maria Smoday, Katarina Oroz, Eva Staresinic, Vilim Dretar, Haidi Yago, Marija Milavic, Suncana Sikiric, Eva Lovric, Lovorka Batelja Vuletic, Paris Simeon, Ivan Dobric, Sanja Strbe, Antonio Kokot, Josipa Vlainic, Alenka Boban Blagaic, Anita Skrtic, Sven Seiwerth, Predrag Sikiric

**Affiliations:** 1 Department of Surgery, School of Medicine, University of Zagreb, 10000 Zagreb, Croatia; 2 Department of Pharmacology, School of Medicine, University of Zagreb, 10000 Zagreb, Croatia; 3 Department of Pathology, School of Medicine, University of Zagreb, 10000 Zagreb, Croatia; 4 Department of Endodontics and Restorative Dentistry, School of Dental Medicine, University of Zagreb, 10000 Zagreb, Croatia; 5 Department of Anatomy and Neuroscience, School of Medicine, J.J. Strossmayer University of Osijek, 31000 Osijek, Croatia; 6 Laboratory for Advanced Genomics, Division of Molecular Medicine, lnstitute Ruder Boskovic, 10000 Zagreb, Croatia

**Keywords:** stable gastric pentadecapeptide BPC 157, muscle healing, therapy

## Abstract

First, we review the definitively severed myotendinous junction and recovery by the cytoprotective stable gastric pentadecapeptide BPC 157 therapy, its healing that might combine both transected and detached tendon and transected muscle, ligament and bone injuries, applied alone, as native peptide therapy, effective in rat injury, given intraperitoneally or in drinking water or topically, at the site of injury. As a follow up, we reviewed that with the BPC 157 therapy, its cytoprotective ability to organize simultaneous healing of different tissues of and full recovery of the myotendinous junction might represent the particular muscle therapy against distinctive etiopathology muscle disabilities and weakness. In this, BPC 157 therapy might recover many of muscle disabilities (i.e., succinylcholine, vascular occlusion, spinal cord compression, stroke, traumatic brain injury, severe electrolyte disturbances, neurotoxins, neuroleptics, alcohol, serotonin syndrome and NO-system blockade and tumor-cachexia). These might provide practical realization of the multimodal muscle-axis impact able to react depending on the condition and the given agent(s) and the symptoms distinctively related to the prime injurious cause symptoms in the wide healing concept, the concept of cytoprotection, in particular. Further, the BPC 157 therapy might be the recovery for the disabled heart functioning, and disabled smooth muscle functioning (various sphincters function recovery). Finally, BPC 157, native and stable in human gastric juice, might be a prototype of anti-ulcer cytoprotective peptide for the muscle therapy with high curing potential (very safe profile (lethal dose not achieved), with suited wide effective range (µg-ng regimens) and ways of application).

## 1. Introduction

This paper attempts to review, in a particular way, the stable gastric pentadecapeptide BPC 157 (for review see, i.e., [1,2,3,4,5,6]) and its effects on striated, smooth, and heart muscles. 

As previously shown (for review, see [1,2,3,4,5,6]), all of the studies to date that have tested the stable gastric pentadecapeptide BPC 157 peptide—native to and stable in human gastric juice, even for periods of time longer than 24 h—as a treatment have demonstrated extremely positive healing effects for various injury types in numerous organ systems, particularly for the muscular system. These might be injuries directly to the muscle or various muscle disabilities deriving from a multitude of causes, peripheral and/or central (for review, see [1,7,8,9]). As an additional effect, there is also the maintenance and recovery of smooth muscle function, and BPC 157 therapy might promote recovery of sphincter functions (for review, see [1,2,3,4,5,6]). As an illustration, it has been shown to counteract tumor-induced muscle cachexia and the signaling process implicated in cancer cachexia [10] and leaky gut [11], as well as its membrane stabilizing and free radical scavenging activities (for review see, [10,11]). Furthermore, its effects on damaged skin, muscle, tendon, and bone are comparable to those in the gastrointestinal tract (and liver, pancreas lesions) [1], kidney and cardiovascular system (particularly affecting blood vessels and vessel recruitment as part of therapy for heart failure and lung lesions, counteracting arrhythmias and thrombosis) [5,6]. Conceptually, its practical significance has been ascribed to its particular role in the Selye’s stress response [3,7,8], as well as to its resolving of activities of the brain–gut and gut–brain axes [7,12]. Of note is the way that BPC 157 might counteract various encephalopathies [13,14,15,16,17,18,19,20,21], behavioral disturbances [22,23,24,25,26,27] (particularly those representing psychiatric illness models [25,26,27]) and CNS disturbance-induced muscle disabilities [19,20,21,22,25,28,29,30,31], in particular. However, the findings that the stable gastric pentadecapeptide BPC 157 might beneficially affect striated and smooth muscle and heart might suggest that it most perfectly matched [32,33,34] with the original Robert’s and Szabo’s cytoprotective theory and concept [35,36,37,38,39,40,41,42]. Originally, the concept holds the epithelium/endothelium protection achieved against direct injury made by noxious agents by contact in the stomach, as direct cell protection, to be translated unlimitedly to the entire body [35,36,37,38,39,40,41,42]. For BPC 157, its essential gastric juice origin and stability in human gastric juice for periods of time longer than 24 h [3,4,32,33,34], due in particular to its special structure (GEPPPGKPADDAGLV), might ascertain the function of the new mediator of cytoprotection. Thus, in the stomach there is the permanent maintenance of mucosal integrity, and thereby in the entire gastrointestinal tract [3,4,32,33,34]. Epithelium/endothelium protection might be easily achieved and further extended to the general level (protection of other organs) (cytoprotection to organoprotection) [3,4,32,33,34]. This implies simultaneous healing of the different tissues (i.e., fistula healing [2], but also myotendinous junction recovery [43]), and thereby particular wound healing potential [1,9], providing a particular potential for the recovery of damaged muscle function [2,19,20,21,22,25,28,29,30,31,43,44,45,46,47,48]. Therefore, BPC 157 has a particular therapeutic effectiveness, including via a therapeutic per-oral regimen, and pleiotropic beneficial effects in terms of cytoprotection [3,4,32,33,34]. Furthermore, BPC 157 might particularly interact with many essential systems, i.e., the nitric oxide (NO)- [49], prostaglandins- [50], dopamine- [26,51,52,53,54,55,56,57], and serotonin- [24,25] systems, known to be essential for both cytoprotection and muscle function integrity, and might interact with many molecular pathways [58,59,60,61,62,63,64,65,66]. Illustratively, this might be the for the control of the vasomotor tone and the activation of the Src-Caveolin-1-eNOS pathway [60,61]. This likely occurred as the particular modulatory effects of the NO-system as whole [49,60,61,67,68]. Indicatively, BPC 157 induced a NO-release of its own [49,67,68] and therefore counteracted both NO-synthase (NOS) inhibition (i.e., N(G) nitro-L-arginine methylester (L-NAME) hypertension and pro-thrombotic effects) and NO overstimulation (L-arginine hypotension and anti-thrombotic, pro-bleeding effects) [49,67,69]. 

Together, this might be a suitable background for a review within the wider frame of the cytoprotection concept [1,3,4,9,32,33,34]. As mentioned, the entirety of BPC 157’s beneficial effect on damaged or disabled muscle function recovery includes the striated and smooth muscle and heart, allowing a new cytoprotective approach to therapy for these muscle disorders. However, standard growth factors are typically rapidly destroyed in human gastric juice, within 15 minutes [1,3,4,32,33,34]. Commonly, these are practical obstacles that cannot be avoided; unable to be applied alone, these growth factors require the addition of various carriers or biological scaffolds [1,70]. 

Furthermore, with BPC 157 therapy, the epithelium/endothelium protection is an innate cytoprotective capability of the agent, and represents, thereby, the essential principle of the cytoprotection principle. Endothelium protection→epithelium protection has been promoted as the particular upgrade of a minor vessel in taking over the function of a disabled major vessel (for review, see [5,6,33]). With severe syndromes, such as vascular and multiorgan failure following major vessel occlusion or similar noxious procedures [66,71,72,73,74,75,76,77,78,79,80,81,82], the particular activation of the collateral pathways (i.e., azygos vein direct blood delivery) might be essential to counteract severe central and peripheral lesions, intracranial (superior sagittal sinus), portal and caval hypertension and aortal hypotension. Likewise, overwhelming thrombosis can be counteracted, and widespread Virchow triad circumstances fully removed [66,71,72,73,74,75,76,77,78,79,80,81,82]. Therefore, the severe muscle weakness that appeared as a decisive outcome was accordingly counteracted as well [80]. 

Thus, particularly following demonstration of the recovery of the myotendinous junction (dissection of quadriceps tendon from quadriceps muscle) [43], this study might provide a particular (cytoprotective) view and evidence of the muscle healing and function recovery with the cytoprotective stable gastric pentadecapeptide BPC 157 therapy [1,3,4,9,32,33,34]. As mentioned, in practice the cytoprotection approach should combine therapy for striated, smooth and heart muscle. Practically, this might be considered a native peptide therapy with high wound healing capacity [1,9] (used without any carrier addition), easily used as a therapy (parenteral, intragastric, per-oral (in drinking water), topical (i.e., cream, solution, eye drops)) [1,3,4,9,32,33,34] and that might also be highly effective in muscle disorders. 

**Figure 1:**
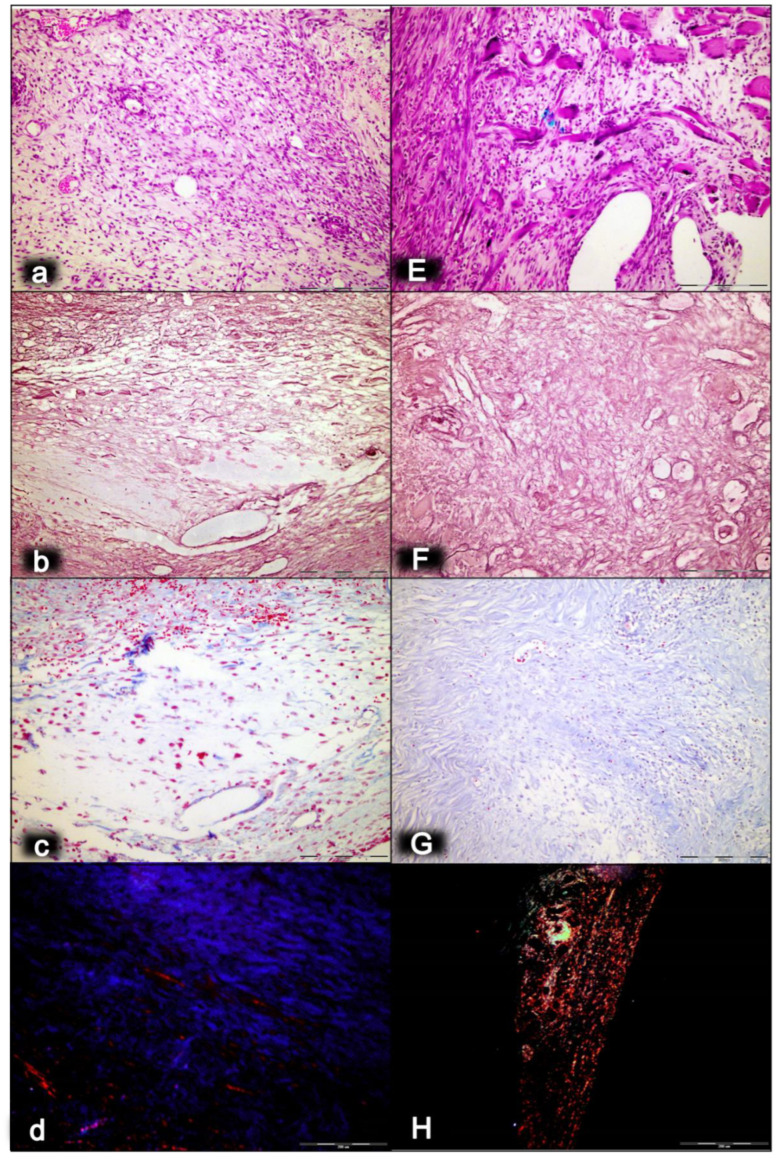
Myotendinous junction seven days following dissection of quadriceps tendon from the quadriceps muscle [43]. Regular presentation after myotendinous junction injury induction in control rats (small letters) (**a**–**d**). Prominent edema, with a marked amount of inflammatory cells, a reduction of capillaries with surviving arterioles of the myotendinous junction (**a**), mash network of crisscrossed reticulin fiber (**b**), and no proliferation of collagen fibers (**c**). Regular presentation after myotendinous junction injury during BPC 157 regimen (capitals), given in drinking water (**E**–**H**). Only mild edema, mild amount of inflammatory cells, and significant vascularity of myotendinous junction with penetrating capillaries (**E**). Pronounced proliferation of fibroblast with production of reticulin and collagen fibers (**F**,**G**). Maturation of the newly formed collagen fibrils in BPC 157-treated rats (**H**) compared with the controls with minimal or no production (**d**) (polarization microscope). HE staining (**a**,**E**); histochemical Gomori staining (**b**,**F**) and Masson trichrome staining (**c**,**G**); Sirius red histochemical staining with polarized microscopy (**d**,**H**); magnification ×200.

**Figure 2:**
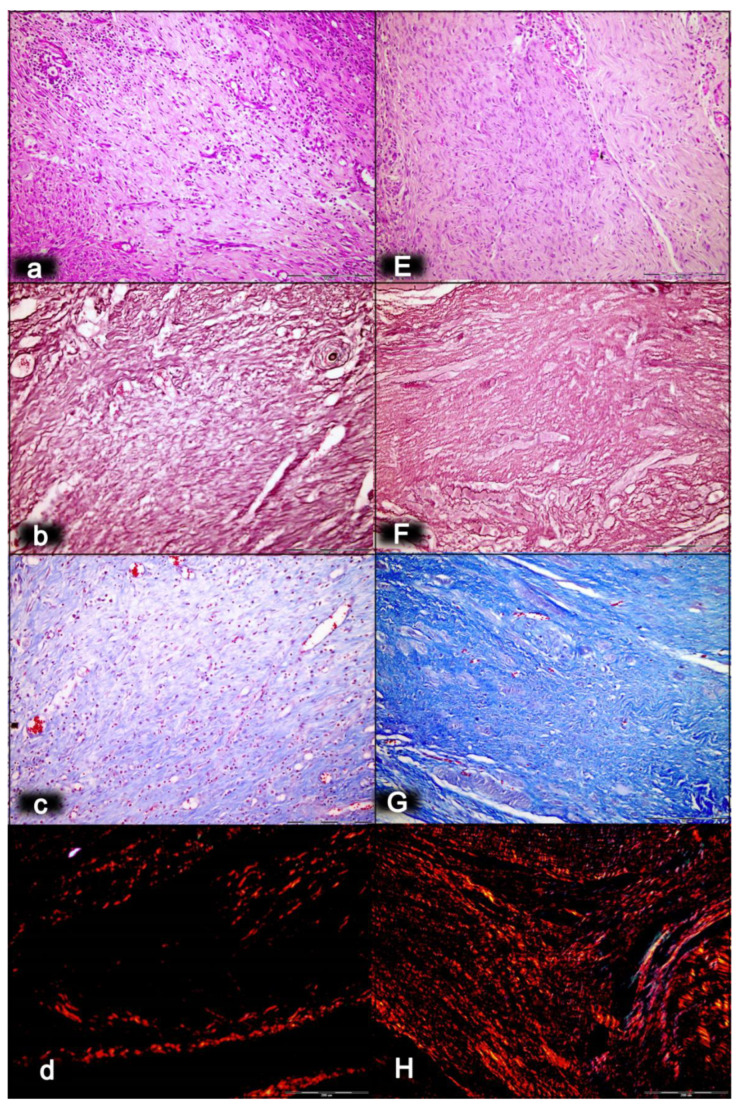
Myotendinous junction 14 days following dissection of quadriceps tendon from the quadriceps muscle [43]. Regular presentation after myotendinous junction injury induction in control rats (small letters) (**a**–**d**). Persistent edema and marked infiltration of inflammatory cells with mild and delayed increase of vascularity of myotendinous junction (**a**). A discrete fibroblast proliferation and reticulin (**b**) and collagen fibers (**c**) production producing a connective tissue with mesh-like fibers and areolar tissue. Regular presentation after myotendinous junction injury during BPC 157 regimen (capitals), given in drinking water (**E**–**H**). No edema and inflammatory cells with well orientated dense connective tissue. Complete vanishing of myotendinous junction revascularization (**E**). Prominent fibroblast proliferation with reticulin (**F**), and collagen fibers synthesis with good orientation and maturation was found (**G**). Using a polarized microscope, sparse and disorganized collagen type 1 fibers were observed in the control (**d**). In treated animals, the appearance of abundant and well-oriented collagen type 1 fibers was found (**H**). HE staining (**a**,**E**); histochemical Gomori staining (**b**,**F**) and Masson trichrome staining (**c**,**G**); Sirius red histochemical staining with polarized microscopy (**d**,**H**); magnification ×200.

**Figure 3:**
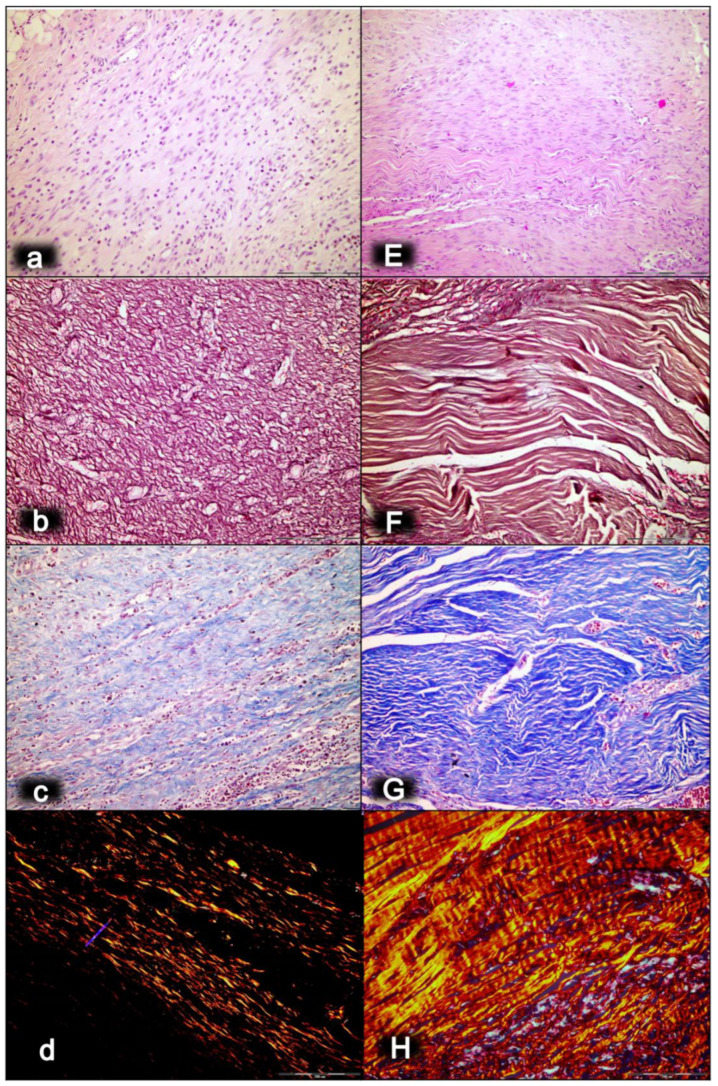
Myotendinous junction 28 days following dissection of quadriceps tendon from the quadriceps muscle [43]. Regular presentation after myotendinous junction injury induction in control rats (small letters) (**a**–**d**). No edema and inflammatory cells were found with vanishing myotendinous junction revascularization (**a**). The proliferation of fibroblasts and fibers, both reticulin (**b**) and collagen fibers (**c**), as well as fiber maturation (much less than in BPC 157 treated rats) with suboptimal orientation to the long axes of the myofibers close to the myotendinous junction. Regular presentation after myotendinous junction injury during BPC 157 regimen (capitals), given in drinking water (**E**–**H**). Well-oriented dense connective tissue was found (**E**). Morphologic features of the myotendinous junction area indicate that BPC 157 therapy favors vascular density as well as reconstruction and orientation of reticulin (**F**) and collagen fibers (**G**). A prominent proliferation of fibroblasts and fibers as well as fiber maturation was obtained (confirmed with polarized microscope; **H**), providing tenable fibroblast and fiber proliferation with optimal orientation due to the long axes of the myofibers close to the myotendinous junction with a lesser number of fibroblasts, and with a higher amount of reticulin and collagen fibers. In contrast, the control group showed far less abundant and well-oriented collagen type 1 fibers (**d**). HE staining (**a**,**E**); histochemical Gomori staining (**b**,**F**) and Masson trichrome staining (**c**,**G**); Sirius red histochemical staining with polarized microscopy (**d**,**H**); magnification ×200.

**Figure 4:**
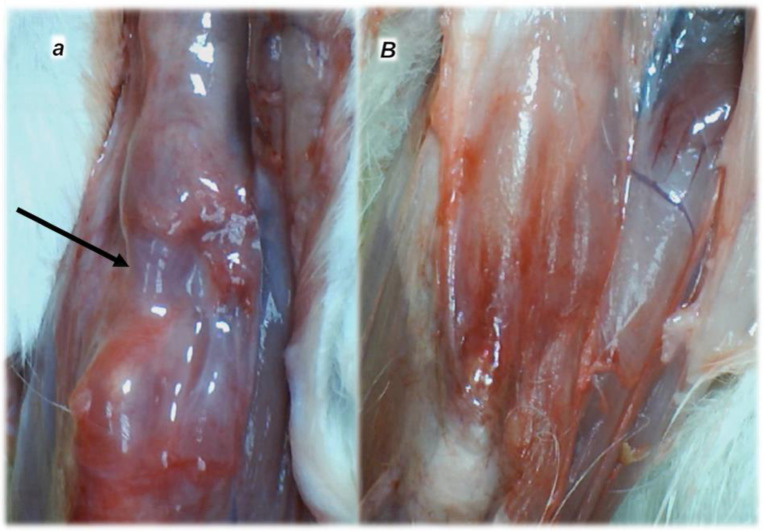
Characteristic presentation of the myotendinous junction healing at six months following dissection of quadriceps tendon from the quadriceps muscle [43] (**a**,**B**). Failed myotendinous junction occurred in the controls (**a**) (gap consequent to dissection of quadriceps tendon from quadriceps muscle (arrow)) and fully reestablished myotendinous junction in the rats treated with BPC 157 (capitals) (10 ng/kg/day orally, in drinking water) (**B**).

**Figure 5:**
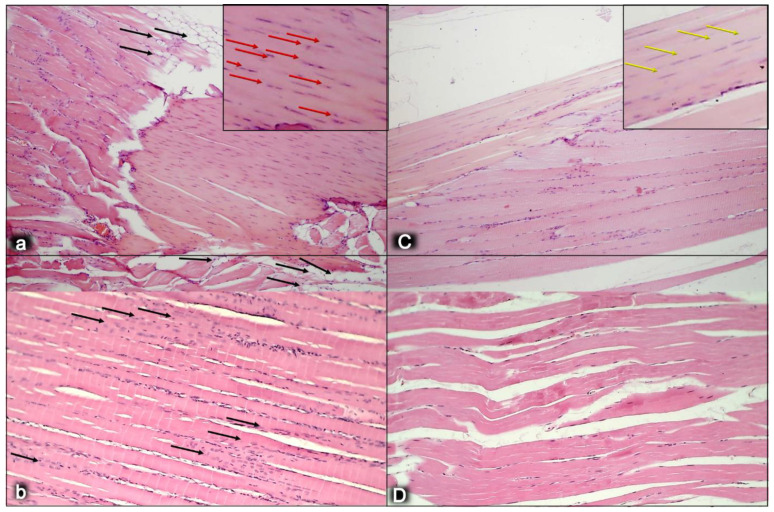
Myotendinous junction six months following dissection of quadriceps tendon from the quadriceps muscle [43]. Regular presentation after myotendinous junction injury induction in control rats (small letters) (**a**,**b**). Tendons have significant numbers of lipid-like structures at the myotendinous junction wound site (black arrows) (**a**). Regular presentation after myotendinous junction injury during BPC 157 regimen (capitals), given in drinking water (**C**,**D**). No lipid-like structures appeared in treated animals (**C**). Also, there is a significantly increased number of typical tendon cells that are more elongated in the treated animals (yellow arrows) (**C**), while there is an increased number of round shaped cells, presumably non-tenocytes, in the control group (red arrows) (**a**). The myotendinous junction is at a high risk for strain injuries, due to the high amounts of energy that are transferred through this structure, indicating the remodeling capacity of the myotendinous junction. The general feature found within a peak period of remodeling is a progressive restoration of the tissue by neosynthesized fibers that are centronucleated. We observed the degree of remodeling of muscle fibers alike the human myotendinous junction in the control group (**b**), where a high portion of the muscle fibers adjacent to the myotendinous junction contained a centrally located myonucleus. This is suggestive of a very high rate of remodeling of the muscle fibers near the myotendinous junction in control group (black arrows) (**b**) in comparison with treated animals (**D**). There were notable differences in the cell kinetics remodeling of fibrils at the myotendinous junction between control and BPC 157 group. HE staining; magnification ×200.

**Figure 6:**
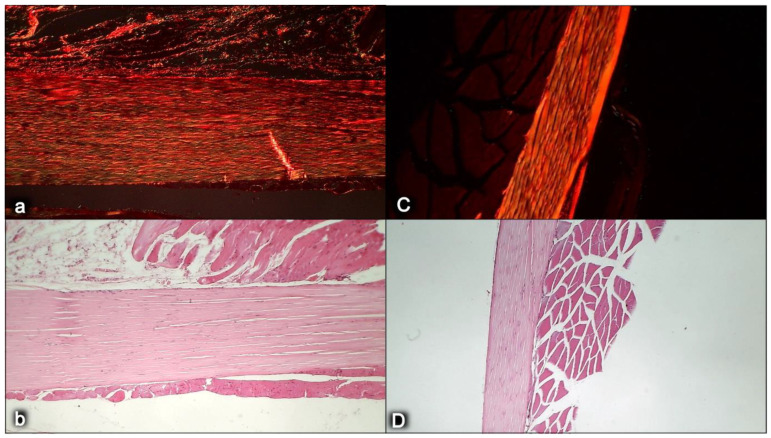
Myotendinous junction six months following dissection of quadriceps tendon from the quadriceps muscle [43]. Comparing the width and length of tendons at six months, we observed shorter and wider tendons in control animals (**a**,**b**) compared with the BPC 157 group (capitals, **C**,**D**). Sirius red staining was conducted to evaluate the maturity of extracellular matrix, especially collagen secretion and production, which is the major component in tendon tissue. The intensity of Sirius red staining was significantly higher in treated animals (**C**) than in control groups (**a**). Polarized light microscopy image showing maturation of the newly formed collagen fibrils in treated animals reaching maturation peak and indicating a marked increase in production of collagen type 1 in contrast to control group. Sirius red histochemical staining with polarized microscopy (**a**,**C**); HE staining (**b**,**D**); magnification ×200.

As an indicative point, the significance of the BPC 157/cytoprotection review for muscle healing functioning is in its resolution of the perception of the cytoprotection complex as a point of interest at the current time, providing more than 2100 studies for “muscle cytoprotection” in Pubmed. This might be perceived as a considerable problem, given that muscle disorders therapy has remained unresolved in general, and that there is neither a conceptual implementation of the original cytoprotection theory nor cytoprotective agents for therapy. On the other hand, the purposeful cytoprotective conceptualization of muscle disturbances with cytoprotective agents might be worthy, given Robert’s and Szabo’s original prostaglandin cytoprotection (stomach) background [35,36,37,38,39,40,41,42], cytoprotection as a commonly acknowledged ongoing physiologic process [83] and prostaglandin E2 as a crucial inflammatory mediator of muscle stem cells and as the building blocks of muscle regeneration [84]. Noteworthily, the concomitant use and mutual counteraction of cytoprotective agents and non-steroidal anti-inflammatory drugs (NSAIDs) has been a common proof of the cytoprotective concept [32,33,34,35,36,37,38,39,40,41,42]. Thus, cytoprotective agents and the cytoprotection concept in general might be suitable for exceedingly common acute muscle injuries. Therefore, the cytoprotective agents and cytoprotection concept in general mandate common acute muscle injuries, NSAIDs to reduce the associated inflammation, swelling and pain, given that NSAIDs prophylactic use, early or delayed administration might delay muscle regeneration and contribute to loss of muscle strength after healing [85]. At the cellular and structural level, evidence exists for a negative influence of NSAIDs on the muscle stem cell population (satellite cells) and on muscle connective tissue’s significant remodeling during muscle regeneration [85]. Furthermore, cardiovascular risk of NSAIDs has appeared to be an under-recognized public health issue [86]. It is important to note that BPC 157, given as therapy, might reestablish prostaglandin system functions, and may promote a counteraction of the adverse effects of NSAIDs [50]. This counteraction might involve the central (i.e., encephalopathies) [15,16,17,18,50], and/or peripheral (i.e., gastrointestinal and liver lesions, bleeding disorders, and muscle disabilities) [15,16,17,18,50,87,88] adverse effects, acting as a membrane stabilizer (counteracted leaky gut) [11] and free radical scavenger, particularly in the vascular studies [10,11,55,66,75,76,79,80,89,90,91] (for illustration, see Figure 1, Figure 2, Figure 3, Figure 4, Figure 5 and Figure 6). 

Thus, in the early 1990s, pentadecapeptide BPC 157 [1,3,4,9,32,33,34,92] appeared as a late offshoot of the cytoprotection–organoprotection concept of Robert and Szabo [32,33,34,35,36,37,38,39,40,41,42], for epithelial and endothelial protection. There is conceptual evidence of BPC 157 (it was found to be distributed in the human gastrointestinal mucosa, lung, bronchial epithelium, epidermal layer of the skin, and kidney glomeruli by in situ hybridization and immunostaining) [1] and its innate activity (i.e., native and stable in the human gastric juice for over 24 h) [1,3,4,5,6,9,32,33,34,92]. Crucially, BPC 157 might act as the particular peptidergic agent that implements the cytoprotective capabilities, and pleiotropic beneficial effects [1,3,4,5,6,9,32,33,34,92] (as noted above, largely involving the muscle healing and function recovery of striated, smooth and heart muscle) [1,3,4,5,6,9,32,33,34,92]. It therefore follows that BPC 157 is a novel cytoprotection mediator, one that is very safe and showed no side effects in clinical trials (i.e., in use for ulcerative colitis), and wherein a lethal dose (LD1) was not achieved in toxicology studies (for review, see [1,9,33,93]). These operational arguments might bring the long-standing theory into practice, starting with the initial argument of the lack of degradation in human gastric juice for periods of time longer than 24 h (for review see, [1,3,4,5,6,9,32,33,34,92]), and thereby its therapeutic effectiveness (including via a therapeutic per-oral regimen) and pleiotropic beneficial effect (for review see, i.e., [1,3,4,5,6,9,32,33,34,92]).

It is likely, in general cytoprotection terms, that this might allow for cytoprotection as a process by which chemical compounds provide protection to cells against harmful agents for the effective healing of the injured muscle and neighboring tissues, as well as for muscle function maintenance and recovery upon either local or systemic disabilities (for review see, i.e., [1,3,4,5,6,9,32,33,34,92]). 

Given its pleiotropic beneficial effects, as part of its cytoprotection background (i.e., simultaneous healing of different tissues) (for review see, [1,5,6,9,33]), there is complimentary recovery of the myotendinous junction as a particular recovery effect of the stable gastric pentadecapeptide BPC 157 [43]. These might represent the general therapeutic effect on the muscle and the healing and functions recovery (and also tendon [58,59,94,95,96,97], ligament [98] and bone [99,100,101]). These might be the therapeutic effects against those induced by either of the direct injuries [43,44,45,46,47,48]. Likewise, these might be the therapeutic effects against those induced by the various muscle disabilities, and the large variety of different noxious events. Given the muscle weakness that is part of the prime disturbance, vascular failure [66,71,72,73,74,75,76,77,78,79,80,81,82], stroke [20], traumatic brain injury [21], spinal cord compression [28,29], vessel occlusion and similar noxious procedures [66,71,72,73,74,75,76,77,78,79,80,81,82] were all specifically highlighted in the literature. Furthermore, the literature also focused on the succinylcholine-induced neuromuscular junction blockade [102], local anesthesia [103,104] (i.e., via lidocaine intraplantar application and axillary and spinal (L4-L5) intrathecal block [103]), electrolytes disturbances [19,55,80,105,106], neuroleptics dopamine blockade [26,51,54,55], NO-system blockade [26], alcohol intoxication [22], serotonin syndrome [25], particular neurotoxins (inducing Parkinson-like disturbances in mice [30] or multiple sclerosis-like disturbances in rats [31]), and tumor cachexia [10]. These intriguing relations might also include, besides the striated muscle, the smooth muscle (i.e., various sphincter functions) [73,91,107,108,109,110,111,112,113,114,115,116,117,118,119,120,121], and the heart muscle [6,54,72,74,76,77,78,79,80,81,82,105,106,121,122,123]. 

Thus, we start with the completely disabled myotendinous junction (for review see, [124,125]) as a focus to be resolved and expanded in further cytoprotection terms [43]. Thereby, the reported myotendinous junction recovery [43] and restoration of full function with the stable gastric pentadecapeptide BPC 157 therapy [43] might have particular therapy potential. This might be taken as the combining healing point of the simultaneously realized recovery of both transected tendon (as well as detached tendon, and osteotendinous junction recovery) [94,95,96,97] and transected muscle [44,48]. In a more extensive way, we should note that a purposive movement requires the impulses passing from the motor cortex via the spinal cord to the appropriate muscles and the movement pattern coordinated by the impulses passing through various parts of the brain and sending messages back to the motor cortex [8]. Thus, the reported myotendinous junction recovery [43] and restoration of full function might illustrate all possible particularities of the capability of the stable gastric pentadecapeptide BPC 157 therapy—applied alone, as native peptide therapy, effective in rat injury, and given intraperitoneally or in drinking water [1,3,4,9,32,33,34].

For the myotendinous junction, as a combination point of anatomical and structural particularities (largely reviewed in [124,125]) such a new therapy possibility might be particularly important given the still unresolved problem of being the weakest element in the muscle–tendon unit, (and bone–tendon junctions as extremely specialized tissues in general). The mechanical utilization of contractile force produced by myofilaments efficiently connected to tendon fibers as a follow up of the BPC 157 therapy was successfully achieved (i.e., recovery of the myotendinous junction and its function following dissection of quadriceps tendon from quadriceps muscle) [43]. Thereby, this BPC 157-induced recovery, consistently achieved with either of its regimens [43], might largely overwhelm the achievement of the current therapy of the myotendinous junction lesions. For the current therapy, the suitable recovery has remained an unachievable point and a particular problem with i.e., biological scaffolds, administration of active compounds, electrospinning, and self-reorganized constructs (for review, see [70]). Commonly, there is lack of delivery or vehicles to localize the factor to the repair site for the relevant period of time and at the appropriate concentration. These might be one of the principal obstacles to the use of cytokines and growth factors in tissue engineering [70]. 

Thus, the finally reported myotendinous junction recovery [43] as novel outbreak may have a general significance in the issue of healing. For general significance, the recovery means that the innate problem was essentially resolved by BPC 157 therapy alone [43]. This means the interrelated muscle and tendon healing— including recovery of the muscle–tendon junction injury and the recovery of the muscle and tendon—occurred simultaneously [43]. Furthermore, pentadecapeptide BPC 157 (unlike transforming growth factor beta (TGF-beta)) has a particular effect on tendocytes in the disturbed conditions [96]. Presenting with no effect on the growth of cultured tendocytes of its own, it consistently opposed 4-hydroxynonenal (HNE), a negative modulator of growth [96]. This finding was also supported by the other studies that confirming BPC 157 promotes the ex vivo outgrowth of tendon fibroblasts from tendon explants and therefore also cell survival under stress [58,59]. Additionally, BPC 157 promoted the in vitro migration of tendon fibroblasts, which is likely mediated by the activation of the FAK-paxillin pathway [58,59]. Likewise, other studies (muscle ischemia [60,61], muscle cachexia [10]) have indicated that particular molecular pathways involvement in the BPC 157 therapy muscle effect [10,11,20,58,59,60,61,62,63,64,65,66]. 

In conclusion, and as mentioned above, as a follow up to the myotendinous junction healing [43], we will further review the significance of these combined points of the BPC 157 therapy effect, the specific healing of the muscle and the function recovery.

## 2. Myotendinous Junction Recovery

In practical principle, myotendinous junction failure occurs when the quadriceps tendon completely tears and the muscle is no longer anchored to the kneecap, so that the quadriceps muscles contract but without function [43]. Thereby, in general, the more complex the injury, the more complex the healing effect that the BPC 157 therapy realized, the more complex are the requirements to confirm the obtained findings. The rats from which we dissected the quadriceps tendon from the quadriceps muscle were continuously monitored throughout a long-term study (42 days). Furthermore, the therapy was fully effective from the very beginning and congruent functional, biomechanical, microscopic and macroscopic assessments consistently support each other. Illustrating the full function as the definitive hallmark of the recovery, regardless of the mechanism, the BPC 157-treated rats had no leg contracture, and no failure to walk (which is otherwise characteristic, wherein, along with the initiation of the swing phase, the foot slides backward as a sudden jerk of the limb towards the back) [43]. The therapy link might also be indicative (note the wide range of the regimen (ng–µg)). It was consistent at each of the investigated post-injury periods and was easily applicable as either an intraperitoneal or per-oral (in drinking water) therapy regimen [43]. More precisely, with the same BPC 157 dose regimen, the myotendinous junction healing [43] occurred alongside the demonstrated restoration of the osteotendinous junction (whereby the Achilles tendon is detached from the calcaneus) and the elimination of the systemic corticosteroid damaging effect [94,95,96,97]. This occurred also as the restored neuromuscular junction function antagonized the effect of the neuromuscular blocker succinylcholine, thereby opposing the inability of the muscle cell to repolarize, and opposing the desensitization at the nerve terminal [102]. Thus, BPC 157 therapy might have a wide but selective healing capacity to restore the disabled junctions and their functions. This might be a healing effect that is particular to the tissue and injury involved. The worst circumstances resolved might be the specific confirmation of the required therapies relating to either transection or detachment of either the tendon or the muscle [44,93,94,95,96,97]. Tendon–tendon continuities were reported to have re-established well, with no ossicles forming in other tissues [94,95,96,97,98] (note, with bone morphogenetic proteins (BMPs) [126,127,128], the initial tendon healing process is misleading, due to its similarity to the process of fracture healing [126] and the formation of ossicles in other tissues [126,127,128]). Likewise, with BPC 157 therapy, there was a re-established muscle–muscle continuity, and thereby a re-established tendon–muscle continuity as well [43,44,45,46,47,48,93,94,95,96,97]. Similarly, this might also occur with a ligament transection, with a reestablished ligament–ligament continuity, and fully recovered function upon medial collateral ligament transection [98]. 

Additionally, given reestablished muscle–tendon, muscle–muscle, tendon–tendon, and tendon–bone continuity [43,44,45,46,47,48,94,95,96,97,98], BPC 157 therapy has considerable bone healing capacity. It heals pseudoarthrosis in rabbits, and femoral head osteonecrosis in rats, it is segmental and counteracts inflammation and alveolar bone loss in experimental periodontitis [32,33,34].

This might be practical evidence that BPC 157 accordingly manages tendon healing and muscle healing [43,44,45,46,47,48,93,94,95,96,97,98], so that myotendinous junction healing may be achieved [43]. Together, the BPC 157 course description (through six weeks) brings a myotendinous junction restoration by BPC 157 as the particular healing course, which is also obviously specific and valuable [43] for the healing other injured tissues, such as muscle, tendon, ligament and bone [32,33,34,43,44,45,46,47,48,94,95,96,97,98], and is probably indicative of other effects as well. Thus, as at no specific point was there a recorded muscle fiber atrophy within the myotendinous area, this is likely a result of the continuously maintained function [43]. Initially, there was significant vascularity, as well as penetrating capillaries, mild edema, infiltration of inflammatory cells, and prominent proliferation of fibroblasts, with the synthesis of the reticulin and collagen fibers of the myotendinous junction. These were later transmitted toward the only well-oriented dense connective tissue, with no edema and inflammatory cells, completely vanished revascularization of the myotendinous junction, and a well oriented dense connective tissue and muscular fibers within the myotendinous junction area [43]. 

Finally, as an indicative hallmark of recovery solely in the disabled myotendinous junction, the BPC 157 therapy showed a suggestive effect. An additional increase of the increased eNOS mRNA level occurred but so did a decrease of the increased COX-2 mRNA levels, as well as a consistently normal level of NO, and a decrease of the increased MDA values almost to the normal level [43]. Thus, a particular interaction with the NO system and prostaglandins system, leading to a counteraction of oxidative stress, occurred [43]. It is likely that BPC 157 specifically acts in conditions of disease as, in the healthy rats, it had no effect. Likewise, myotendinous junction healing as an effect of oxidative stress and stress on the NO and prostaglandin systems [43] might be approached with BPC 157 as a particular modulation of the activities of the NO and prostaglandins systems, and as a counteraction to the oxidative stress [49,50,60,61,67,68,69]. These were observable as the spontaneous release of NO [67,68], as a counteraction of the adverse effect of a NOS blockade (i.e., L-NAME hypertension and pro-thrombotic effect), a counteraction of the adverse effect of NOS overstimulation (i.e., L-arginine hypotension and anti-thrombotic effect) [67,69], as control of vasomotor tone and as the activation of the Src-Caveolin-1-eNOS pathway [60,61]. There was also evidence of the maintenance of the thrombocytes function (i.e., without interfering with coagulation pathways) [69,87,88], the counteraction of all adverse effects of NSAIDs [50], and the role of membrane stabilizer (counteracting leaky gut) [11] and free radical scavenger, particularly in the vascular studies [10,11,55,66,75,76,79,80,89,90,91] (for illustration, see Figure 1, Figure 2, Figure 3, Figure 4, Figure 5 and Figure 6).

In summary, the myotendinous junction healing and its further applicability might be seen as realization of the particular wound healing effect of BPC 157 as particular cytoprotective agent (for review, see [1,3,4,5,6,9,32,33,34,92]). The advantage of the native peptide therapy is its combination of both local and systemic effectiveness, avoiding all problem associated with the need for carriers. Most importantly, there is clear evidence of the effect, which is contrary to the peptide–carrier complex (for review, [1,3,4,5,6,9,32,33,34,92]). Thus, we can claim that the myotendinous junction recovery [43] occurred alongside the described beneficial effect in the healing of the muscle [43,44,45,46,47,48] and the tendon [94,95,96,97,98]. There were consistent functional, biomechanical, macroscopic, and microscopic effects for the exemplified mechanism(s) that allowed the definition of myotendinous junction healing in practice [43]. Therefore, the functional recovery, muscle size recovery, and oxidative stress may be particularly illustrative [43]. This might also be seen in further prolonged studies (Figure 4, Figure 5 and Figure 6).

## 3. Muscle Healing

In BPC 157 studies, in addition to the myotendinous junction (dissection) [43], muscle lesions were induced by transection, contusion, corticosteroid application, and nerve transection [44,45,46,47,48]. It is evident that the effect of BPC 157 therapy might cover distinctive aspects of the lesions arising from the given variety of the applied injury (transection vs. contusion vs. denervation) [43,44,45,46,47,48]. Local (transection, contusion) or close (denervation, close nerve transection) injuries might serve as examples for the successful healing of the directly injured muscle [43,44,45,46,47,48]. Furthermore, evidence in the rats has so far shown the recovery of the various muscle disabilities deriving from a multitude of different causes, peripheral and central, given that muscle weakness was part of the primary disturbance that was also attenuated. The multitude that appeared to be covered and thereby ameliorated by the suggested cytoprotective activity includes vascular failure [66,71,72,73,74,75,76,77,78,79,80,81,82], stroke [20], traumatic brain injury [21], spinal cord compression [28,29], vessel occlusion and similar noxious procedures [66,71,72,73,74,75,76,77,78,79,80,81,82]. Furthermore, counteraction occurred with the succinylcholine-induced neuromuscular junction blockade [102], and local anesthesia [103,104] (i.e., via lidocaine intraplantar application and axillary and spinal (L4-L5) intrathecal block [103]). Similarly counteracted were the consequences of electrolytes disturbances [19,55,80,105,106], neuroleptics dopamine blockade [26,51,54,55], NO system blockade [26], particular neurotoxins (inducing Parkinson’s-like disturbances in mice [30] or multiple sclerosis-like disturbances in rats [31]). Furthermore, alcohol intoxication [22], and serotonin-syndrome [25] were counteracted. Finally, tumor-cachexia was counteracted [10]. Additionally, the reported evidence included the maintenance and recovery of the smooth muscle function (i.e., various sphincter functions) [73,91,107,108,109,110,111,112,113,114,115,116,117,118,119,120,121], and the heart muscle [6,54,72,74,76,77,78,79,80,81,82,105,106,121,122,123]. Thus, in general, this resolved multitude might be the result of the multimodal muscle–axis impact. This might be able to react depending on the condition and the given agent(s) as well as on the symptoms that are distinctive to the primary cause of injury in the wider healing concept. In this case the novel and important point might be the implementation of the concept of cytoprotection (for review, see [1,3,4,5,6,9,32,33,34,92]). Thus, for the BPC 157 therapy, the translation to the preserved muscle function might consistently occur as a well-functioning cytoprotection-loop (i.e., brain-periphery) (for review, see [1,3,4,5,6,8,9,32,33,34,92]). 

Moreover, the recent findings might be helpful to resolve the interconnected entirety of a disabled muscle’s problems, as the initial or the end point. In this, and in the face of the advance of severe occlusion syndrome, and occlusion-like syndrome following major vessel occlusion or other similarly noxious procedures [66,71,72,73,74,75,76,77,78,79,80,81,82], there has been recent BPC 157 therapy demonstrations of the rapid activation of the collateral pathways related to the injury [5,6,66,71,72,73,74,75,76,77,78,79,80,81,82]. Thereby, as a part of the counteraction of the symptoms of severe vascular and multiorgan failure syndrome, and of progressing Virchow counteraction, there was counteraction of severe lesions of the brain, heart, lung, liver, kidney and gastrointestinal system, progressing thrombosis, in peripheral and central arteries and veins [5,6,66,71,72,73,74,75,76,77,78,79,80,81,82], intracranial (superior sagittal sinus), portal and caval hypertension, and aortal hypotension, all of which were attenuated or eliminated. For multimodal muscle axis combining the multiple targets involved in resolving the muscle injury and maintaining function this might be a well-functioning cytoprotection loop (i.e., brain-periphery), where an upgrading of a minor vessel compensates and takes over the function of the disable major vessel and reestablishes the reorganized blood flow [5,6,66,71,72,73,74,75,76,77,78,79,80,81,82]. This was taken as an effective upgrade of the cytoprotection principle of endothelium maintenance → epithelium maintenance [5,6,66,71,72,73,74,75,76,77,78,79,80,81,82] (note, long ago, the principle of cytoprotective agents’ pleiotropic beneficial activities postulated the founding of two major groups of (stomach) studies: Robert (epithelium maintenance) and Szabo (endothelium maintenance) [35,36,37,38,39,40,41,42] (for review, see [32,33,34]). Thus, BPC 157, as a powerful cytoprotective agent, might rapidly act to recruit collateral pathways. Specifically, the recovery of the general muscle weakness in the lithium-intoxicated rats, whatever the initial or end point, might be indicative of the counteraction of the lithium intoxication as whole [80]. This might be the indicative proof of the general concept for the recovery from muscle disability (counteracting the stasis of peripheral and central thrombosis, with a direct effect on the maintenance of the thrombocytes function but without affecting coagulation pathways) [5,6,66,71,72,73,74,75,76,77,78,79,80,81,82]. Thus, the muscle weakness recovery assumed the recovery from the multiorgan failure and the recovery from the particular central and peripheral vascular failure [66,71,72,73,74,75,76,77,78,79,80,81,82]. This vascular recovery effect characterized the azygos vein, as the rapidly upgraded minor vessel to take over the function of the disabled major vessel, to compete with the ongoing injury, and to bring direct blood flow to the superior caval vein [66,71,72,73,74,75,76,77,78,79,80,81,82]. This was promptly associated with the recovery of the severe muscle weakness and with the recovery of the severe heart disturbances and the rapid reduction of brain swelling [80]. Similar counteraction of severe muscle weakness occurred in rats with abdominal aorta anastomosis, marked attenuation of the vessel obstruction (early regimen), and full annihilation of the obstructing thrombus (late regimen) [129].

Thereby, this wide cytoprotection agenda (i.e., the cytoprotective agents’ direct (epithelial) cell protection is transmitted from the stomach to offer a similar beneficial effect in other organ lesions (cytoprotection → organoprotection)) [35,36,37,38,39,40,41,42], with application of BPC 157 therapy (for review, see [1,3,4,5,6,8,9,32,33,34,92]), might be distinctive and might overwhelm the focused background of the current pharmacotherapy. Furthermore, the beneficial effects ascribed to the BPC 157’s muscle healing and muscle function recovery, might have a very wide range (for review, see [1,3,4,5,6,8,9,32,33,34,92]). In its full extent, the range of BPC 157 therapy and its huge curing potential as a “treatment” (for review, see [1,3,4,5,6,8,9,32,33,34,92]) might largely override the range of the beneficial effects commonly reported with standard cytoprotective agents (for review, see [35,36,37,38,39,40,41,42]) (i.e., prostaglandins’ beneficial effects on the stomach [35], intestine [38], liver [130], pancreas [39], kidney [38,131], and heart [132]). Likewise, its easy applicability during the course of an injury’s treatment might bypass the limitation of the standard agents (for review, see [1,3,4,5,6,8,9,32,33,34,92]) (i.e., the greater effect normally thought to result from “prevention” than “treatment” [35,36,37,38,39,40,41,42]). Moreover, with the rapid endothelium maintenance as the key for a pleiotropic beneficial effect, there is a particular emphasis on the capacity of a large wound to heal itself [1,9], due to both its epithelium and endothelium integrity maintenance capabilities.

By contrast, while fibroblast growth factor (FGF) potential to induce skeletal muscle angiogenesis has been extensively investigated (for review see, i.e., [133]), the practical significance of such FGF, and even vascular endothelial growth factor (VEGF) [134], for muscle healing means an effect that is mostly limited to direct local administration combined with different modes of delivery [135]. Evidently, far from providing conclusive evidence, one peptide and numerous carriers (thereby, diverse peptide + carrier(s) complexes) might create particular problems (though there is uncertain attribution of the data, as noticed by Marshall Urist, discoverer of the activity of BMPs in the 1960s [136]). Consequently, there might be a variation in the healing evidence related to the diverse carriers and delivery systems (epidermal growth factor (EGF), FGF, VEGF, BMPs)) (for review, see [1,9]).

**Figure 7:**
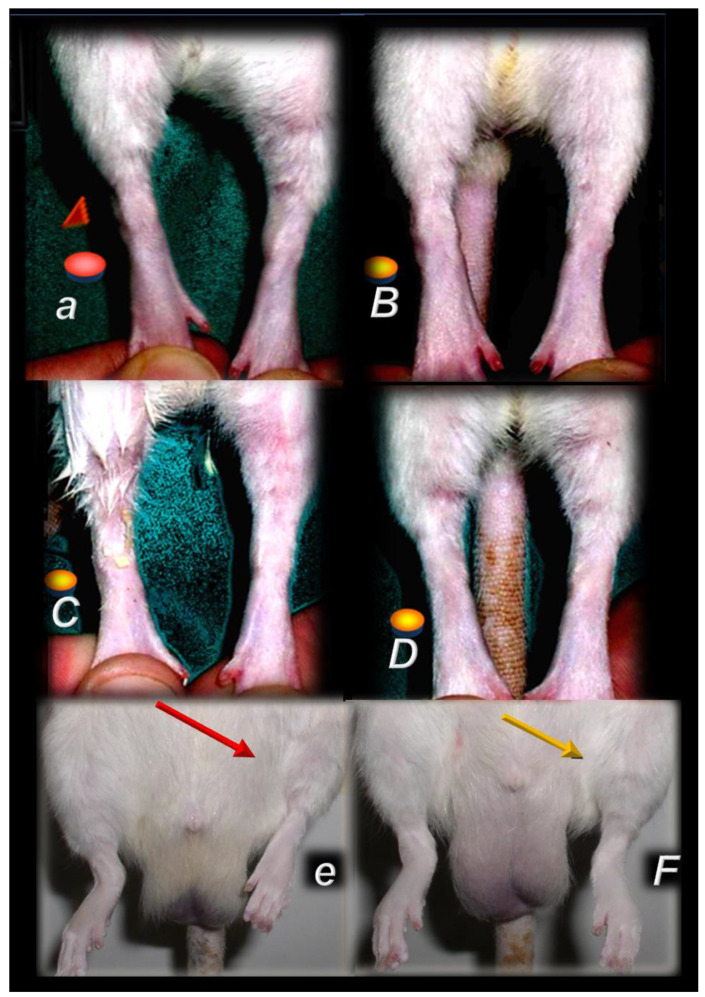
Injured leg contracture, prominent in the controls (small letters), following muscle transection at 72 days post-injury [44] (**a**) or denervation at one year [47] after injury (**e**), or, in contrast, fully counteracted in pentadecapeptide BPC 157 treated rats (capitals) (quadriceps muscle transection (**B**–**D**), gracilis muscle denervation (**F**)). In the rats, which underwent major muscle transection, leg contracture in controls upon maximal extension (red circle, red triangle) (**a**). Contrarily, BPC 157 rats presented no contracture of the injured leg (yellow circle) in the BPC 157 rats. The following BPC 157 regimens were used: per-orally, 10 ng/kg, in drinking water, 0.16 ng/mL; 12 mL/rat/day (**B**), locally with cream (1 µg/g neutral cream, thin layer once daily) (**C**), and intraperitoneally 10 µg/kg/day (**D**). Illustrative presentation of the injured leg at one year after denervation (cut obturator nerve), presentation of injured leg (rats in up-right position) with spontaneous contracture (red arrow) in control rats (**e**). Contrarily, injured leg presented without contracture with BPC 157 (10 ng/kg/day, intraperitoneally) (yellow arrow).

In addition to the mentioned rapid effect on the activation of the collateral vessels and the reestablished blood flow [66,71,72,73,74,75,76,77,78,79,80,81,82] that are part of the essential cytoprotection agent’s activity, BPC 157’s healing effect might exert a strong angiogenic effect as a particular effect, which overwhelms that of the standard antiulcer agents [1,9,137]. Indicatively, BPC 157 promoted angiogenesis in chorioallantoic membrane assay and tube formation assay [61]. Accordingly, BPC 157 might accelerate the blood flow recovery and vessel number in rats with hind limb ischemia [61]. This was attributed to the up-regulation of VEGFR2 expression in rats with hind limb ischemia and endothelial cell culture, as well as promotion of VEGFR2 internalization in association with VEGFR2-AKT-eNOS activation [61]. It is worth noting that this prominent angiogenic effect in the healing of the injured muscle might be closely linked to the advanced improvement of the healing effect (shift toward the left) and is likely specific to the tissue being healed [48]. Indicatively, BPC 157 might consistently promote avascular tissue healing in the tendon (i.e., myotendinous junction, transected/detached tendon healing) [43,93,94,95,96,97,98] and cornea (BPC 157 heals corneal ulceration in rats and maintains corneal transparency [138,139]), with specificity for the preservation of corneal avascularity, as “angiogenic privilege”, essential for corneal wound healing [140]. It is likely that this might ascertain a balance between the proangiogenic and antiangiogenic mediators [138,139,141]. As an additional advantage, BPC 157 itself has also showed a prominent anti-tumor effect [10,142] and might counteract the VEGF-tumor promoting effect [142], as well as tumor cachexia [10]. For illustration, see Figure 7. 

Also, as mentioned above, BPC 157’s healing effect has been found to act as a membrane stabilizer (counteracting leaky gut syndrome) [10] and a free radical scavenger [10,11,55,66,75,76,79,80,89,90,91]. These effects have been recently associated with several molecular pathways [10,11,20,58,59,60,61,62,63,64,65,66], in particular with the increased expression and internalization of VEGFR2, and the activation of the VEGFR2-Akt-eNOS signaling pathway [60,61], interacting particularly with the NO [49] and prostaglandins systems [50]. 

### 3.1. BPC 157 and Muscle Healing and Muscle Function

*Transection*, *Crush*, *Denervation*

*Nerve Injury*

It should be stated that the therapeutic effect on the used rat muscle transection (i.e., quadriceps muscle completely transected transversely 1.0 cm proximal to patella) might be smoothly translated (definitive defect, 72-day period) [44]. This might be claimed also for the regimen (the first application 30 min post-transection, the final 24 h before sacrifice) of the given BPC 157 therapy. The given dose range (10 µg, 10 ng, 10 pg/kg intraperitoneally, once daily) might ascertain the suited wide therapy range, and easy therapy application for each of the included parameters. The biomechanic function, microscopy/immunochemistry, and macroscopy improvement consistently supported each other. Advanced healing of the otherwise irreparable defect included quite extensive assessment. The load of failure increased and walking recovery and extensor postural thrust/motor function index returned toward normal healthy values. Most muscle fibers were connected to muscle segments with no gap and significant desmin positivity for the ongoing regeneration of muscle. Both sides, distal and proximal, presented the larger myofibril diameters and reached normal and healthy rat-values. The stumps were approximately connected and subsequent atrophy was markedly attenuated, finally, they presented as performing similarly to a normal non-injured muscle, with no post-surgery leg contracture [44].

There might be a similar interpretation of the therapeutic effect in the crushed rats (crushed gastrocnemius complex, 0.727 Ns/cm^2^, 2 cm proximal to the Achilles tendon insertion) [45,46]. Assessed as described above, the immediate and long-lasting recovery occurred with BPC 157 given intraperitoneally or locally as a cream (i.e., less hematoma and edema, better walking, no post-injury leg contracture, almost no scar tissue). Force delivered 0.727 Ns/cm^2^ might provide a considerable lesion. Likewise, BPC 157’s therapeutic healing effects occurred to ameliorate against all adverse effects related to the crush injury as external mechanical pressure, including the counteraction of the increased enzyme activity (creatinine kinase, lactate dehydrogenase, aspartate aminotransferase, alanine aminotransferase) [45]. Muscle proteolysis after local trauma was also thereby counteracted [45]. This BPC 157 effect (given in the same regimen, intraperitoneally or locally as a thin layer of cream) was also persistent in the rats treated daily with 6-alpha-methylprednisolone intraperitoneally [46]. Considering the described counteraction of the systemic corticosteroid-impaired healing (i.e., tendon injuries [94,95,96,97] and burns [143,144,145]), BPC 157, given either intraperitoneally or locally, completely eliminated the damage induced by systemic corticosteroid application in rats with crushed gastrocnemius complex [46]. Evidently, it maintained its undisturbed therapeutic effect (note, BPC 157 locally administered as 1.0 μg or 0.01 μg dissolved in distilled water per gram of commercial neutral cream corresponded to the therapy effect observable in the severely burned animals [143,144,145]). Furthermore, BPC 157 eliminated systemic corticosteroid treatment functionally, macroscopically, and histologically at all investigated intervals [46].

**Figure 8:**
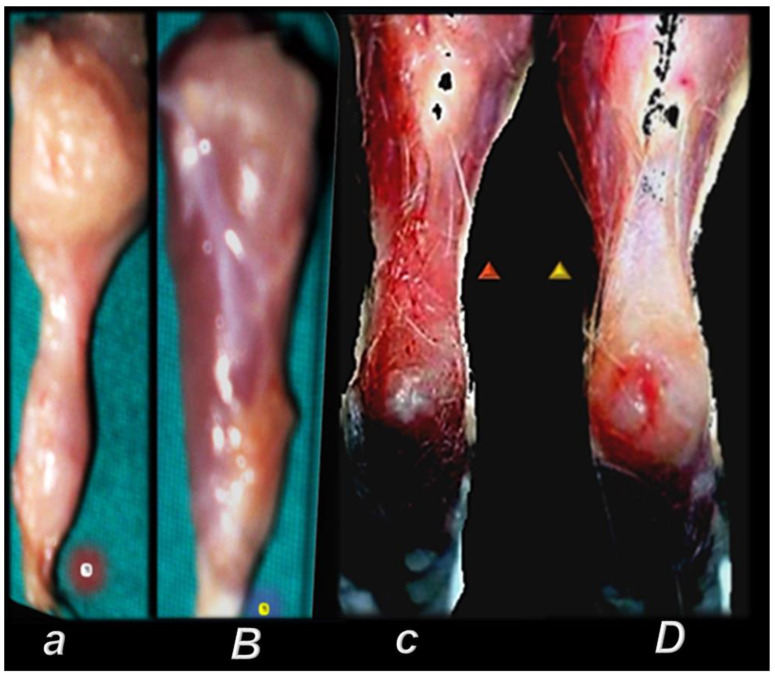
Gross presentation of the perilous course after major muscle transection (quadriceps muscle (circle indicates distal part of the muscle) (**a**) [44], and after the rat’s Achilles tendon was sharply transected from the calcaneal bone (**c**) [94] (triangle indicates significant gap between the tendon edge and bone with a clear stump). Injurious course (controls, small letters) was counteracted by pentadecapeptide BPC 157 (capitals). BPC 157 regimens (10 µg, 10 ng, 10 pg/kg, intraperitoneally, once daily) improved gross presentation (transected muscle, muscle presentation with regeneration and absent marked atrophy, at day post-surgery day 72 (**B**); detached tendon, no defect between the tendon stump and calcaneal bone. The edge of the tendon stump cannot be recognized (osteotendon junction re-established (**D**)), and there is functional, biomechanical microscopical, immunohistochemistry healing improvement.

**Figure 9:**
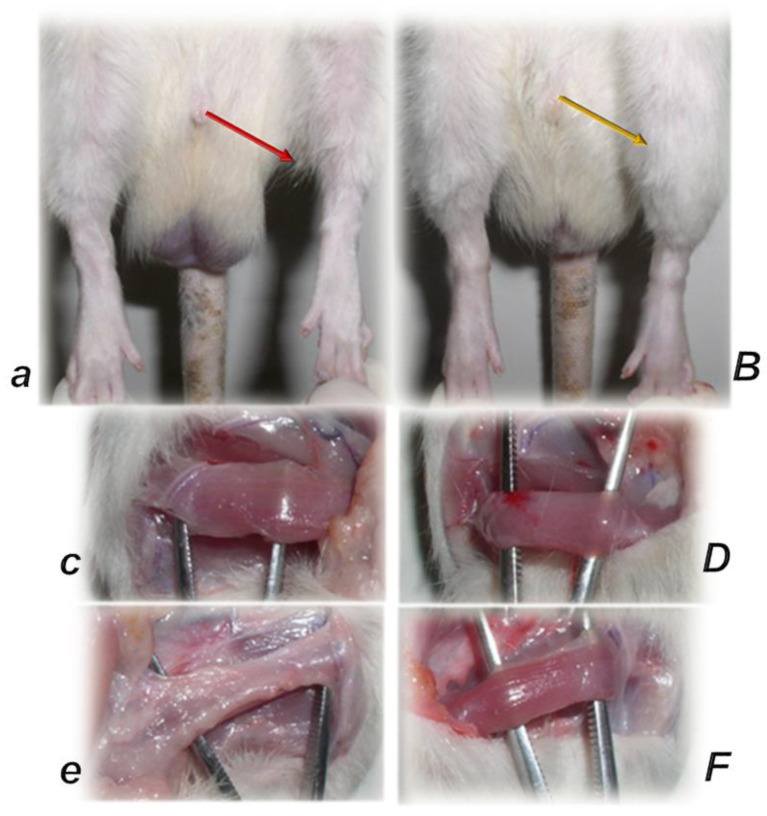
Pentadecapeptide BPC 157 (capitals) prevented muscle atrophy and preserved muscle function after denervation in one year study (controls, small letters) [47]. Contracture upon maximal extension (rats in up-right position), control, left, injured leg contracture (red arrow) (**a**); BPC 157, right, injured leg without contracture (yellow arrow) (**B**). Gracilis muscle: control, left: non injured leg, non denervated (**c**), injured leg, denervated muscle (**e**); BPC 157, right: non injured leg, non denervated (**D**), injured leg, denervated muscle (**F**). Microscopy controls presented many smaller muscle fibers with centralized nuclei in muscles while BPC 157 presentation was not morphologically different from the healthy group.

After denervation, pentadecapeptide BPC 157 therapy counteracted muscle atrophy and preserved muscle function from at least one year [47] (Figure 7, Figure 8 and Figure 9). Gracilis muscle denervation induced considerable atrophy (shorter diameter of muscle fibers, no more than 70% of that of the non-injured leg, smaller muscle weight, many smaller muscle fibers with centralized nuclei), failed function (decreased muscle function index and tottering walk) and a post-injury leg contraction [47]. This was completely reversed in BPC 157 rats throughout the experiment in all of the pentadecapeptide BPC 157 regimens. The same muscle fiber diameters and muscle weight in injured and non-injured (healthy) legs, morphologically the same as the healthy legs, appeared as a consistent outcome of both BPC 157 regimens (10 µg/kg or 10 ng/kg intraperitoneally per day) [47]. 

Considering the motoneuron regeneration, BPC 157 rats exhibited faster axonal regeneration as both local and general effect [146]. In particular, they presented improved presentation of neural fascicles, homogeneous regeneration pattern, increased density and size of regenerative fibers, existence of epineural and perineural regeneration, uniform target orientation of regenerative fibers, and a higher proportion of neural vs. connective tissue. All fascicles in each nerve showed increased diameter of myelinated fibers, thickness of myelin sheet, number of myelinated fibers per area and myelinated fibers as a percentage of the nerve transected area and the increased blood vessels presentation. The absent autotomy accords with the advanced healing, both electrophysiologically (increased motor action potentials) and functionally (improved walking as improved sciatic functional index (SFI)) [146]. In these terms, the evidence regarding both local and general effect confirms that the healing effect might be initiated at any time. The application was shortly after anastomosis injury, intraperitoneal or intragastric or local, at the site of anastomosis. Likewise, the application was later inserted into the tube with the non-anastomosed nerve after a 7-mm segment had been resected [146].

Additionally, considering the motor function recovery and given the recovery following local anesthetic lidocaine [103] and the neuromuscular blocker succinylcholine [102], BPC 157 might simultaneously act at the muscle, neuromuscular junction, and at the nerve, on both sides.

BPC 157 therapy might counteract the effect of the lidocaine-induced local anesthesia via intraplantar application, and axillary and spinal (L4-L5) intrathecal block [103]. Illustratively, given as an early or late therapy, BPC 157 therapy might quickly induce in axillary-block full recovery of the failed function (otherwise, there is a long-lasting inability to walk, and grasp) and limb edema elimination. Illustratively, given as an early or late therapy, BPC 157 therapy might, in the spinal block, quickly induce full recovery of the failed hind limb function (otherwise, there is a prolonged hind limbs failure, flaccid paralysis with no motor recovery for 90 min) [103]. Furthermore, it seems that BPC 157 therapy might affect the effect of the lidocaine as whole. Specifically, BPC 157 might counteract the lidocaine-induced arrhythmias and convulsions as well as lidocaine-induced HEK293 cell depolarization [103].

Finally, BPC 157 therapy might protect the rat’s somatosensory neurons both against capsaicin neurotoxicity and after capsaicin damage and might help to regain the somatosensory neuron function [147,148]. Additionally, BPC 157 increased the survival rate of cultured enteric neurons and the proliferation rate of cultured enteric glial cells (EGCs) [64]. In principle, the increased survival of cultured enteric neurons and the proliferation of cultured EGCs may improve healing of damaged enteric nervous and mucosal structures. This was claimed as the cytoprotective mechanism and application of BPC 157 in potential therapy for enteric neural injury and gastrointestinal ulcers [64].

### 3.2. Muscle Disabilities after Succinylcholine Application

Given the muscle functioning in general, the maintained function of the neuromuscular junction [102] is likely to be essential for the realization of the multimodal muscle-axis impact’s ability to react depending on the condition and the given agent(s) and the symptoms distinctively related to the primary cause of injury within the wider healing concept, particularly the concept of cytoprotection. Given succinylcholine, the counteraction of the widespread disabilities of the neuromuscular junction in the muscles [102], might be the implementation of the concept of the cytoprotection (for review, see [1,3,4,5,6,8,9,32,33,34,92]). Namely, the cytoprotection agent might widely protect cells against direct injury produced by the direct contact of a given noxious agent (for review, see, [35]). In this context, the succinylcholine directly induced a blockade of the neuromuscular junction [102]. Thus, the succinylcholine effect [102] might appear as the prime example of the direct injury to muscle and muscle function, even more direct than the trauma-induced muscle lesions [43,44,45,46,47,48]. The concept was that BPC 157, given before or immediately after succinylcholine, counteracted succinylcholine effects [102]. Counteraction might include a local paralytic effect in the injected muscle or immediate leg contracture (also presented long after systemic muscle disability has abated) [102] (note, it was seen that chronically denervated muscles develop contracture and that the acetylcholine receptors develop over the entire surface of denervated skeletal muscle fibers after their motor nerve has been severed [149]). The initial agitations before muscle disability, the countless muscle twitches before complete muscle tonus loss, and the motionless laying were all counteracted [102]. Thereafter, the hyperalgesia, violent screaming, pain upon light touch, muscle fibers decrease, and edema in the injected and non-injected quadriceps muscle and diaphragm at 1, 3, 5, and 7 days after intramuscular succinylcholine were all counteracted [102]. Thus, it seems that the succinylcholine effects were attenuated, directly and/or indirectly (i.e., single action potential in adjacent membrane after binding, while succinylcholine remains bound there, keeping the end plate potential depolarized). Additionally, the hyperkalemia, arrhythmias and rise in serum enzyme values in the succinylcholine-rats were counteracted with BPC 157 therapy [102].

The final argument might provide the comparable beneficial effect of the two BPC 157 regimens, intraperitoneal application or per-oral application in drinking water, either before succinylcholine, or immediately after succinylcholine, in a considerable dose range [102]. Together, the consistent beneficial effect of BPC 157 might mean the common effect of an adequate maintenance of both the acetylcholine receptor function and neuromuscular junction function. This is in spite of succinylcholine application and is consistently achievable with distinctive BPC 157 regimens [102]. 

### 3.3. Further Defining BPC 157/Muscle Relation

#### 3.3.1. Muscle Disabilities after Vascular Failure

For the suggested multimodal muscle axis impact activation, in particular against the ongoing vascular failure, there was a recovery of the rats disabled with abdominal aorta anastomosis [129]. This might be the given hallmark for the particular relation of the muscle function preservation/recovery and the cytoprotection endothelium integrity maintenance/recovery (for review, see [1,3,4,5,6,8,9,32,33,34,92]). This might rapidly contribute to the counteraction of the otherwise obligatory and severe muscle disability. Namely, without therapy, the rats with abdominal aorta anastomosis exhibited continuous severe painful muscle disability (either spontaneous or induced vocalization), very weak muscle strength and tottering walk, or even full inability to walk [129]. The therapy outcome was quite consistent. The harmful development was avoided (or at least attenuated) via an early BPC 157 regimen. Confronted with the full and advanced development of harm, late BPC 157 regimen induced rapid counteraction. These beneficial effects accord with thrombus development attenuation (early regimen), or elimination (late regimen) [129]. Given the marked attenuation of the initial thrombus development, walking that was slow but still normal and without tottering—and even a normal walk at optimal speed with no tottering—was a characteristic result of the BPC 157 therapy given as a bath at the abdominal anastomosis immediately after surgery [129]. Furthermore, with the late BPC 157 therapy, wherein the thrombus was annihilated, walking quickly recovered. The time point for BPC 157 therapy (intraperitoneal) was at 24 h post-surgery in the rats that had firmly established thrombus and full vessel occlusion [129].

A more complex cause–consequence relation might appear in the stroke rats, with the therapy following removal of the bilateral clamping of the carotid arteries, assessed at 24 h and 72 h of the reperfusion [20]. There were full functional recoveries (Morris water maze test (preserved spatial learning and memory), inclined beam-walking test (preserved locomotor capability), and lateral push test (preserved resistance to a lateral push from either side of the shoulder)). These were along with the counteractions of both early and delayed neural hippocampal damage in the BPC 157 rats [20]. mRNA hippocampal expression studies at 1 and 24 hr provided additional support. There was strongly elevated (*Egr1*, *Akt1*, *Kras*, *Src*, *Foxo*, *Srf*, *Vegfr2*, *Nos3*, *and Nos1*) and decreased (*Nos2*, *Nfkb*) gene expression (*Mapk1* not activated). It is likely that these might fully indicate BPC 157 therapy as a particular activity [20]. From the brain cytoprotection viewpoint (for review, see [8,12]), and particular loop functioning upon BPC 157 administration, this integral effect (i.e., preserved brain and muscle function), consistently achieved with the BPC 157 therapy, might be particularly important [20]. The standard cytoprotective therapy exhibited only an incomplete effect [150]. There was structural brain integrity preservation during the acute post-occlusion period (decreased infarct size) by standard cytoprotective therapy (i.e., calcium channel blocker, isradipine) [150]. However, this therapy effect remained without preservation of the function [150]. Moreover, as additional support, there was a consistent beneficial effect of the BPC 157 therapy in mice after traumatic brain injury [21]. This might be particularly indicative in terms of both direct injury and vascular impact, and the respective cytoprotection implementation. There was considerable counteracting potential against instant unconsciousness (absence of the righting reflex), and death, and the subsequent deleterious outcomes of prominent brain edema, hemorrhagic traumatic lacerations, and subarachnoidal and intraventricular hemorrhage [21]. Thus, the preserved righting reflex with traumatic brain injury might be taken to substantiate that BPC 157 therapy might promptly counteract the immediate consequences as well as attenuate and improve the otherwise deleterious outcomes [21]. 

An even more complex cause–consequence relation might be provided by BPC 157 therapy in the reperfusion with the recovery of the tail paralysis after spinal cord compression [28,29]. Rat studies covered the long post-injury period (one year), and, in particular, the immediate period following application, given at the time of the early or late course of the spinal compression. Spinal cord compression-induced tail paralysis was irreparable in the untreated rats [28,29]. Contrarily, there was a consistent recovery achieved with the BPC 157 therapy, given at once, soon after injury (i.e., at 10 min post-injury), or much later (i.e., therapy at fourth post-injury day) [28,29]. The regimen in which the therapy was applied once effectively counteracted the regular presentation of the cysts and the loss of axons instead of hemorrhagic areas in the white matter. Together, throughout the entire long period, the sustained tail motor score debilitation and sustained tail paralysis and spasticity were counteracted (which would otherwise present until the end of the experiment (day 360)). Similarly counteracted were the decreases in the number of large, myelinated axons in the caudal nerve, and the higher motor unit potential (giant potential) in the tail muscle [28]. Indeed, an important part of the muscle function recovery and a successful outcome of BPC 157 therapy, has been the rapid attenuation of the spinal cord hematoma and rapid disappearance of the swelling observed in a further detailed study and directly observed at a point 10 min after injury [29]. At 30 min post-injury, microscopically, the lesion was reversed to only a discrete edema with minimal hemorrhage [29]. Accordingly, there were increased Nos1, Nos2, and Nos3 values [29]. Moreover, the same recovering effect on the disabled muscle function occurred with a much later therapy application. A similarly rapid therapeutic effect occurred in the protracted spinal cord injury (four days post-injury, BPC 157 intragastric application) [29]. Again, microscopic examination revealed the reversal to only mild hemorrhage, and only discrete vacuolation of tissue (at 20 min following therapy application at day four post-injury). In addition to the acute recovery, rats which had definitive tail paralysis might have comparable long-term recovery with the per-oral therapy in drinking water, beginning at day four after injury and lasting one month thereafter till the end of the experiment [29]. BPC 157 rats rapidly presented tail function recovery with no demyelination process (Luxol fast blue staining) [29]. 

Finally, given the lithium intoxication as a syndrome [80] that presented similarly to those induced by major vessel(s) occlusion and other similar procedures [66,71,72,73,74,75,76,77,78,79,80,81,82], the recovery from severe muscle weakness in rats repeatedly treated with the high dose of the lithium chloride might be directly and promptly combined with the achieved vascular recovery [80]. This is a specific effect (upgrading of a minor vessel to take over and compensate the function of a failed major vessel, re-establishing the reorganized blood flow). As a particular cytoprotection action, it might closely combine the multitude of the muscle function recoveries with the resolution of the recovered concomitant vascular failure [80]. To perceive the significance of the BPC 157 therapy and the activation of the collateral pathways [66,71,72,73,74,75,76,77,78,79,80,81,82], it should be emphasized that, without therapy, the lithium treated rats experienced severe muscular weakness, failed and congested vessels, and full occlusion-like syndromes [80]. Along with muscular weakness, there was progressing intracranial (sinus sagittal superior), portal and caval hypertension, and aortal hypotension, progressing thrombosis, in arteries and veins, both peripherally and centrally, severe ECG disturbances, and severe lesions in the brain, heart, lung, liver, kidney and gastrointestinal tract [80]. These were all counteracted by application of BPC 157 therapy [80]. Illustrating that such competition (i.e., the rapidly upgraded minor vessel takes over the function of the disabled major vessel) is effective against the ongoing injury as whole [66,71,72,73,74,75,76,77,78,79,80,81,82], it might be essential that the recruited activated azygos vein brings a direct blood flow to the superior caval vein. Consequently, the recovery of the severe muscle weakness and severe heart disturbances might be promptly associated [80]. 

Thereby, we suggest that this vascular recovery might serve as a common defensive principle [5,6,66,71,72,73,74,75,76,77,78,79,80,81,82]. It might also be applicable to the recovery from muscular disabilities. Namely, similar vascular and multiorgan failure presentations (and counteractions with BPC 157 therapy) have been seen with other similar occlusion syndromes and occlusion-like syndromes, after occlusion of the major vessel(s), arteries and veins, both peripherally and centrally, and with similar noxious procedures. This particular beneficial effect may be competing with the Virchow’s triad, something that is a common explication [66,71,72,73,74,75,76,77,78,79,80,81,82].

Furthermore, these BPC 157 effects [20,21,28,29,80,129] should be considered as its particular and successful effect on wounding (i.e., abdominal aorta anastomosis [129] vs. amputation of the leg or tail [66,88], counteraction of obstructing thrombus formation and rapid annihilation of fully established obstructing thrombus [129] vs. decreased post-amputation bleeding [66,88]). Thus, we claim that the realized healing effects in the various wounds (for review see, i.e., [1,9]) might confirm the realized healing process for ruptured blood vessels as whole. Thereby, it might have this innate distinctive effect on all four major events in clot formation and dissolution, and might be used in distinctive ways depending on the given injury and agent application. Such a special effect might be highly applicable and could contribute to the relief of various muscular disturbances [20,21,28,29,80,129].

#### 3.3.2. Muscle Disabilities with Systemic Electrolytes Disturbances

Commonly, given muscle functioning in general, finding the maintained muscle function despite severe electrolyte disturbances [19,55,80,105,106], whatever their cause, should be essential given that the impact of the multimodal muscle axis might react depending on the condition and the given agent(s) and the symptoms distinctively related to the primary cause of injury. Indeed, strongly effective BPC 157 therapy regimens in both lithium and potassium overloaded rats shared the same high lithium [80] or potassium [105] serum concentration as the control lithium and potassium overloaded rats [80,105]. Likewise, effectiveness in the hypokalemic rats (overdose of furosemide) [106] follows the low potassium serum level corresponding to the those in the control potassium depleted rats [106]. Thus, these BPC 157 effects may reveal an intriguing point in that BPC 157 therapy seemed to overwhelm electrolyte disturbances (including muscle disabilities) in rats [19,55,80,105,106] as part of its cytoprotective effect on the tissue target(s) (for review, see [1,3,4,5,6,8,9,32,33,34,92]). This might ascertain a relation of close-to-normal functioning with high-serum lithium or potassium, or low-serum potassium [105,106], rather than the disturbed function that may be expected. Consequently, BPC 157 was strongly effective against all of the electrolyte disturbance-induced muscular weaknesses and against all other disorders, which were investigated depending on the focus of the particular study and were perceived in rats with an excess of the hyperkalemia, hypokalemia, hypermagnesemia and hyperlithemia [19,55,80,105,106]. Note that the used regimens were accommodated to the overdose of potassium (9 mEqu/kg ip) [105], magnesium (560 mg/kg ip) [19] and lithium (500 mg/kg ip/3 days) [80], and furosemide (100 mg/kg ip) [106] or succinylcholine (1.0 mg/kg into the right anterior tibial muscle) [102]. Thereby, in these conditions, the therapy was given to counteract the severe disturbances (including muscular weakness in particular), or even lethal outcomes [19,55,80,105,106]. In the potassium overdose-induced excessive hyperkalemia (>12 mmol/L), BPC 157 therapy, given before or late in the already advanced hyperkalemia, might counteract the lethal outcome (that would without therapy have occurred within 30 min) [105]. Likewise, it might counteract the muscular weakness, severe arrhythmias, hypertension, low pressure in the lower esophageal and pyloric sphincters along with mucosal lesions when the stomach was directly exposed to overwhelming potassium overwhelming [105]. Furthermore, in the furosemide overloaded rats, and severe hypokalemia (without therapy, fatal within 150 min), BPC 157 therapy ascertained survival [105]. As early treatment, it maintained the sinus rhythm and eliminated the presentation of the arrhythmias, and myoclonus. As the late treatment, given in the hypokalemic rats presented with third grade AV block and/or ventricular tachycardia, within a minute it would normalize ECG, terminate ventricular tachycardia, and eliminate myoclonus [105]. 

Magnesium intoxication was seen as acting as a loop to translate hypermagnesemia, brain lesions and muscle lesions to the severe muscle weaknesses and prostration, in a very short time (minutes) [19]. BPC 157 therapy might counteract magnesium overdose as whole. The hypermagnesemia and hyperkalemia, severe muscle weakness and prostration, decreased muscle fibers in both the quadriceps muscle and the diaphragm, increased serum enzyme values, and nerve damage and edema in various brain areas were additionally counteracted. The most prominent damage in the cerebral cortex was particularly counteracted [19]. 

The counteraction of lithium overdose by BPC 157 therapy has the largest loop that was assessed to be translated to severe muscle weakness and prostration, whatever the cause–consequence relations [80]. Along with fully counteracted severe muscle disturbances, there were brain, heart, lung, liver, kidney and gastrointestinal tract lesions, along with progressing venous and arterial thrombosis progressing stasis, both peripherally and centrally, all of which were counteracted by BPC 157 therapy application. Along with the counteracted ECG disturbances, intracranial (superior sagittal sinus) hypertension, portal and caval hypertension, and aortal hypotension were eliminated/attenuated [80]. 

It is likely that the effect of BPC 157 on potassium overdose, furosemide overdose, magnesium overdose, evidenced in vitro (HEK293 cells), might have an indicative significance and that this might be generalized. There may be BPC 157 effects on K+ conductance and a particular effect on membrane potential [105,106]. BPC 157 alone was able to depolarize HEK293 cells. To a much higher extent, BPC 157 reduced depolarization induced by hyperkalemic conditions (as well as depolarization induced by bupivacaine [122], lidocaine [103], or magnesium-overdose [19]). Thus, BPC 157 particularly decreases the cell membrane conductance for potassium during hyperkalemic conditions (or high depolarizing activity of the other agents) [105]. On the other hand, similar counteracting effects were obtained in the hypokalemic condition [106]. This might convey the furosemide overloaded hypokalemic rats (˂2.7 mmol/L) with the simultaneous counteraction of the adverse effects in the cardiac muscle and in the skeletal muscle [106]. In principle, decreased extracellular potassium entails myocardial hyperexcitability, with the potential to develop re-entrant arrhythmias in the skeletal muscle, hyperpolarization of the resting membrane potential, and a greater-than-normal stimulus for depolarization of the membrane in order to initiate an action potential [106]. 

**Figure 10:**
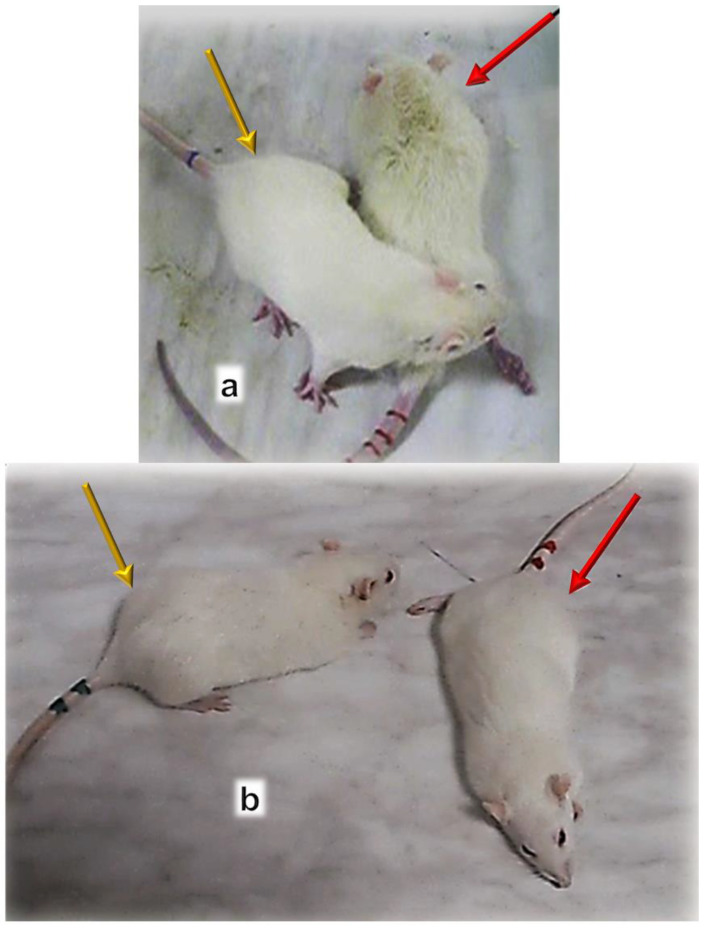
Muscle disability after abdominal aorta anastomosis [129] (**a**). Muscle disability with hypermagnesemia induced by magnesium sulfate (560 mg/kg intraperitoneally) (**b**) [19]. Abdominal aorta anastomosis (**a**). The illustrative control animal unable to walk (red arrow), at 24 h after abdominal aorta anastomosis creation, and illustrative normal walk preserved (BPC 157 given as bath at the abdominal anastomosis immediately after surgery), or quickly recovered (BPC 157 given intraperitoneally at 24 h post-surgery) (yellow arrow). Hypermagnesemia (**b**). Illustrative presentation of control rat, severe muscle weakness leading to complete muscle disability and inability to move (red arrow). BPC 157 treated rats (10 µg, 10 ng/kg intraperitoneally at 15 min before magnesium) continuously maintained a normal appearance, undisturbed muscle function (yellow arrow).

Thus, there might be particular relations between the skeletal muscles (i.e., the largest single pool of K^+^ in the body [151]) and BPC 157 therapy. It is likely that these particular relationships might be indicative of the strongly recovered healing and skeletal muscle function upon various injurious events, trauma [43,44,45,46,47,48] and non-direct trauma [10,20,21,26,28,29,51,54,55,66,71,72,73,74,75,76,77,78,79,80,81,82,102,103,104] induced disturbances. These healing effects might have a considerable role in balancing the interconnected hyperkalemia/hypokalemia (i.e., hyperkalemia (i.e., exercise) is rapidly corrected by reaccumulation of potassium into the muscle cells via Na^+^, K^+^ pumps, often leading to hypokalemia [151]). This might be regarded as the essential cytoprotective principle (direct cell protection)—constant cell function preservation whatever the injurious effect (for review, see [1,3,4,5,6,8,9,32,33,34,92]). On the other hand, with BPC 157 therapy for the counteracted lithium-overdose-induced severe muscle disturbances, as the principle applied to other electrolytes disturbances, there was a large range of concomitantly counteracted multiorgan failures and lesions [80]. The brain, heart, lung, liver, kidney and gastrointestinal tract lesions were counteracted, as were the venous and arterial thromboses and stasis was abolished, both peripherally and centrally, along with the counteracted ECG disturbances [80]. Likewise, BPC 157 therapy eliminated/attenuated intracranial (superior sagittal sinus) hypertension, portal and caval hypertension, and aortal hypotension [80]. Thereby, the rapidly upgraded minor vessel was able to take over the function of the disabled major vessel. The azygos vein offered direct delivery to the superior caval vein, which might generally be associated with the recovery from severe muscle weakness and severe heart disturbances and might be the key to compete with the ongoing injury in other electrolyte disturbances as well [80]. For illustration, see Figure 10. 

#### 3.3.3. Muscle Disabilities with Disabled Dopamine, Serotonin and NO-System

It might be suggested that the impact of BPC 157 therapy is in accordance with the significance of the dopamine system, commonly acknowledged to control muscle function [8,12] (and also, since the very beginning, implemented in the cytoprotection concept [152,153]). Thereby, in general terms, the particular importance of the agent might be suggested when it might affect either of the dopamine system functions, inhibitory or stimulatory. The most important point occurs when the agent, such as BPC 157 therapy, might have a modulatory effect that might affect both functions and might provide the needed balance of both functions [8,12]. Furthermore, considering the muscle disabilities that may be recovered with the dopamine-system–BPC 157 therapy interactions, BPC 157 might have a complex therapy effect. BPC 157 largely interacts with the dopamine system (a topic reviewed specifically elsewhere [8,12]) since BPC 157 counteracts various disturbances, tremors, akinesias, and catalepsies that appear within the dopamine system disability [26,30,51,52,54,55,56,57]. BPC 157 therapy counteracted disturbances caused by 1-methyl-4-phenyl-1,2,3,6-tetrahydrophyridine (MPTP)-application and destruction of brain dopamine areas [30]. Likewise, BPC 157 counteracted disturbances caused by reserpine application and thereby vesicle depletion [30]. BPC 157 counteracted disturbances of the dopamine receptor blockade (that occurred in the course of the haloperidol, fluphenazine, clozapine, and sulpiride applications [26,30,51,52,54,55,56,57]), both peripherally and centrally. On the other hand, BPC 157 may counteract the opposite disturbances (i.e., stereotypies) that would appear in the course of the amphetamine, methamphetamine or apomorphine, or dopamine (over)-stimulation [26,52,53]. Note, these effects were assessed as counteractions of the positive-like symptoms and negative-like symptoms in the models of schizophrenia [26,27].

Confronted with severe serotonin syndrome, gastric pentadecapeptide BPC 157 ( with no behavioral or temperature effect of its own) has a beneficial activity, which is likely to be specific and related to a rather specific counteraction of 5-HT2A receptors phenomena [24]. Serotonin syndrome commonly follows irreversible monoamine oxidase (MAO)-inhibition and subsequent serotonin substrate (in rats with fore paw treading, hind limbs abduction, wet dog shake, and hypothermia followed by hyperthermia) [24]. This counteracting effect might be a specific effect. Namely, in depression models, BPC 157 therapy reduced the duration of immobility to a greater extent than imipramine [23]. This is likely to be different from any other serotonergic drug as BPC 157, given peripherally, has a region-specific influence on brain serotonin synthesis (alpha-[14C]methyl-L-tryptophan autoradiographic measurements) in rats [25]. Indicatively, it might particularly induce serotonin release in the substantia nigra [25] that might be important for the maintenance of muscle function with BPC 157.

A similar conclusion might also be given for the functioning of the NO system given that BPC 157 in the same dose range might fully counteract catalepsy induced by the NOS-blocker L-NAME [26].

## 4. BPC 157 Effect on Smooth Muscle Functioning

It might be that the particular effect of BPC 157 in electrolyte disturbances (i.e., hyperkalemia, hypokalemia, hypermagnesemia, and hyperlithemia [19,55,80,105,106]), and membrane potential [19,103,105,106,122], might confirm the particular effect of BPC 157 on the smooth muscle, and vice versa. Particular sphincters (lower esophageal sphincter, pyloric sphincter [107,108,109,110,111,112,113,114,115], pupil [73,116], urinary sphincter [91,117,118]) with distinctive functions, are all affected in the same way during conditions of sickness and the recovery of disabled function. This particular effect might suggest a distinctive therapy effect depending on the injury condition, along with the general content of the cytoprotection concept (i.e., maintained cell integrity against different noxious agents’ injurious effects) (reviewed elsewhere, see [1,3,4,5,6,8,9,32,33,34,92]). Additionally, it might maintain conditions of the sphincter’s normal functioning, with a modulating effect on distinctive sphincter functions. As illustration, these might be an anti-reflux effect (i.e., BPC 157 increased lower esophageal sphincter pressure, and decreased pyloric sphincter pressure [107]), the maintenance of normal pupil diameter [116], or the maintenance of normal leak point pressure [117]. The indicative recovery from sickness conditions, might consistently highlight the recovered function of the disabled sphincters (i.e., recovery of the decreased pressure within lower esophageal sphincter and pyloric sphincter [107,108,109,110,111,112,113,114,115]; counteraction of the prolonged pupil mydriasis or miosis [73,116], counteraction of the decreased leak point pressure [91,117,118]). This maintenance or recovery of sphincter function by BPC 157 therapy might be contrary to different agents’ application, (i.e., absolute alcohol, NSAIDs and/or neuroleptics and/or NO-agents, and/or atropine (lower esophageal sphincter, pyloric sphincter, pupil) [107,108,109,110,111,112,113,114,115,117], cyclophosphamide (urinary sphincter) [91], and different (even opposite) dysfunctions, i.e., NOS-blockade, and NOS-over-activity [55,113]). Additionally, similar maintenance or recovery of the sphincter function by BPC 157 therapy might be against different noxious procedures. These include stress urinary incontinence after transabdominal urethrolysis and prolonged vaginal dilatation [116], glaucoma induction (episcleral veins cauterization) [73], esophagitis (tube insertion into sphincter(s)) [107,108,110], acute pancreatitis (bile duct ligation) [110], creation of fistulas [111,112,118], and, in particular, anastomosis creation [115]. The sphincter recovery consistently goes along with the prime causative lesion recovery and healing [55,73,91,107,108,109,110,111,112,113,114,115,116,117,118]. Illustratively, in rats with esophagogastric anastomosis [115], at the site of anastomosis and along with anastomosis healing, BPC 157 therapy promptly realized a “sphincter-like function” and efficiently substituted the original sphincter function. Specifically, it might simultaneously recover the pyloric sphincter function (that is otherwise irreparably failed and thought to reflect the failure of the lower esophageal sphincter’s function) [115]. Functionally, in severe cyclophosphamide hemorrhagic cystitis lesions (i.e., urothelial necrosis, vesical edema, erosion, hemorrhage, inflammation, and ulceration, increased bladder weight), the increased leak point pressure was reversed to the values noted in the normal rats [91]. Additionally, the BPC 157 therapy might fully reverse the otherwise detrimental course after prolonged vaginal dilatation or transabdominal urethrolysis and might recover the decreased leak point pressure to normal values to ameliorate the rat’s stress urinary incontinence (likely analogous to human injury [117]). A similar effect may be seen in rats with vesicovaginalis fistula, i.e., macro/microscopic recovery, counteracted stone formation, and functional recovery (elimination of vaginal leaking—represented by the ability to maintain a five-fold-larger volume before leaking—in comparison with controls) [118]. In the eye, counteraction of the presentation of glaucoma with BPC 157 therapy in rats with cauterized episcleral veins was exhibited through normal pupil diameter (eliminated mydriasis), well preserved ganglion cells and optic nerve presentation, normal fundus presentation, normal retinal and choroidal blood vessel presentation and normal optic nerve presentation [73].

Finally, BPC 157 therapy might exert the described specific effects on smooth muscles in vivo as specific therapy effects. The evidence might be the relaxation noted in the aorta without endothelium ex vivo as opposed to the lack of relaxation directly on the 3-D model composed of vascular smooth muscle cells (unlike the effect of NO-donor sodium nitroprusside) [61]. This evidence might suggest the additional specific points to be considered for the demonstrated spontaneous release of NO [49,67,68], activated phosphorilazation of eNOS [61], counteraction of the adverse effect of NOS-blockade (i.e., L-NAME hypertension and pro-thrombotic effect), and counteraction of the adverse effect of NOS overstimulation (i.e., L-arginine hypertension and anti-thrombotic effect) [49,67,69]. The VEGFR2-Akt-eNOS signaling pathway might be activated without the need for other known ligands or shear stress, controlling vasomotor tone and the activation of the Src-Caveolin-1-eNOS pathway [60,61]. These might also be perceived as BPC 157-specific mechanisms, supplemental to direct NO stimulation. In this respect, we should also view the maintenance of the thrombocyte function (i.e., without interfering with coagulation pathways) [69,87,88], as well as the BPC 157 interaction with NSAIDs and the counteraction of all adverse effects, as the recovery of the prostaglandin system [50]. Additionally, and as mentioned above, BPC 157’s role as a membrane stabilizer (counteracting leaky gut) [11] and free radical scavenger, particularly in vascular studies [10,11,55,66,75,76,79,80,89,90,91], might be particularly beneficial effects. 

## 5. BPC 157’s Effect on Heart Function

For additional definition of the BPC 157–muscle relation, the complex effect of BPC 157 therapy on heart function may be interesting (for review, see [6,7]). The particular effects of BPC 157 on the maintenance of the NO system’s function [49] resulted in the BPC 157 therapy promptly reversing already established doxorubicin chronic heart failure [121]. Supporting this observation were the effects of BPC 157 medication on serum endothelin values when BPC 157 rescued doxorubicin-related cardiac failure [121]. Counteracted increments of the endothelin values went along with the reversal of the heart failure, the reversal of the increased values toward normal levels was similarly concomitant with the cardiac failure reversal [121]. 

Very recently, in isoprenaline-rats, the stable gastric pentadecapeptide BPC 157 appeared as a useful peptide therapy against isoprenaline myocardial infarction, as well as against isoprenaline myocardial reinfarction [79]. Isoprenaline course appears to follow the early full multiorgan failure developed as an occlusion/occlusion-like syndrome [66,71,72,73,74,75,76,77,78,79,80,81,82], induced peripherally and centrally, and first time described in the isoprenaline rats [79]. The leading role of rapid heart disturbances, and thereby, the heart, lung, liver, kidney and gastrointestinal lesions, leads to inferior caval vein and superior mesenteric vein congestion; azygos vein failure, portal and caval hypertension and aortal hypotension; central intracranial (superior sagittal sinus) hypertension; brain swelling and severe lesions; and widespread thrombosis [79]. BPC 157’s therapy effect was ascribed to the activation of the additional collateral pathways (i.e., azygos vein to provide direct blood delivery, i.e., inferior caval vein–azygos vein–superior caval vein rescuing pathway) [79]. Following the therapy was a consistent counteraction of early and full multiorgan failure (and heart failure, in particular) and thrombosis of the described occlusion/occlusion-like syndrome [66,71,72,73,74,75,76,77,78,79,80,81,82], peripherally and centrally, and thereby, counteraction of the later isoprenaline-infarction and isoprenaline-reinfarction [79]. 

Moreover, BPC 157 therapy has a rapid effect on heart disturbances in similarly complex and deleterious circumstances in all of the studies of the permanent occlusion of major vessel-induced occlusion syndromes [66,67,68,69,70,71,72,73,74,75,76], as well as occlusion-like syndromes induced by severe intoxication (alcohol, lithium) [80,81], permanent bile duct occlusion [78] and intra-abdominal hypertension [77]. Recovery was via the activation of the collateral pathways to compensate vascular failure [66,71,72,73,74,75,76,77,78,79,80,81,82], and there is compelling evidence that BPC 157 therapy realized both prophylactic and curative effects. Furthermore, in lithium rats the reversal of the severe muscular weakness was promptly combined with the restoration of cardiac function [80].

BPC 157 therapy counteracted various arrhythmias. These included arrhythmias induced by digitalis overdose [154], hyperkalemia [105], hypokalemia [106], succinylcholine [102], bupivacaine [122], lidocaine [103], neuroleptics [133], major vessel occlusion [66,67,68,69,70,71,72,73,74,75,76], major intoxication (alcohol, lithium) [80,81], permanent bile duct occlusion [78], maintained intra-abdominal hypertension [77], and hypoxic injury and reoxygenation arrhythmias [123]. There was a particular reference on the counteraction of the prolonged QTc interval (i.e., as a class effect of neuroleptics [54]), observable also in the BPC 157 therapy prevention and reversal of monocrotaline-induced pulmonary hypertension in rats [82].

Thus, the final result of the additional determination of the BPC 157–muscle relation might be the innate ability of BPC 157 to aid in heart failure recovery as a whole [7]. This might include the counteraction of various arrhythmias [66,67,68,69,70,71,72,73,74,75,76,77,78,80,81,82,102,103,105,106,122,123,154] and thromboses, even blood pressure disturbances (intracranial (superior sagittal sinus), portal and caval hypertension, and aortal hypotension [66,71,72,73,74,75,76,77,78,79,80,81,82], or hypertension (hyperkalemia, NOS-blockade) [67,105] that were attenuated/eliminated peripherally and centrally)). Therefore, the complex effect of BPC 157 therapy on heart functioning may be interesting (for review see, i.e., [6,7]).

## 6. BPC 157 and Cuprizone Neurotoxin (Mimicking Multiple Sclerosis-like Disturbances in Rats)

A further highlight would be the counteraction of the complex severe muscle disability that was induced by the neurotoxin cuprizone in rats [31]. Cuprizone-induced demyelination in animals is commonly accepted for studying multiple sclerosis-related lesions and is characterized by degeneration of oligodendrocytes more than by a direct attack on the myelin sheet [31]. Thus, in order to resolve the principle behind cuprizone’s adverse effect, and muscle disability in particular, we argue for a direct demonstration of the healing and the recovered functionality of the severely injured muscles by trauma injury [43,44,45,46,47,48] or otherwise [10,20,21,26,28,29,51,54,55,66,71,72,73,74,75,76,77,78,79,80,81,82,102,103,104], combined with the healing of the severely injured nerves [146,147,148]. The other studies indicate a beneficial effect on various encephalopathies [13,14,15,16,17,18,19,20,21], not all of which relied on immunomodulation. By contrast, there is no similar study on the healing effect of disease-modifying drugs on the severely injured muscles and severely injured nerves [31]. BPC 157 was highly effective when used similar regular regimens (10 μg or 10 ng/kg, per-orally/intragastrically) [31] and shown to be effective in all BPC 157 studies (for review, see [8,12]).

To accentuate all the disturbances, and to emphasize the obtained therapeutic effectiveness, we used a cuprizone regimen several times higher than those regularly used in cuprizone studies [31,155]. This protocol rapidly induced marked functional disability, in particular, a difficulty with maintaining body balance while rearing, an inability of the right forelimb to react (and thereby cuprizone-rats react only with one or no forelimb), and severely affected various brain areas [31]. Contrarily, BPC 157 rats do not spare the right forelimb, reacting simultaneously with both forelimbs to grasp the forceps, maintaining body balance while rearing, and showing less nerve damage—particularly in those areas that had been most affected (corpus calosum, nucleus reuniens, anterior horn motor neurons) [31].

## 7. BPC 157 and Muscle Wasting in Tumor-Cachexia

BPC 157 therapy effectively counteracted muscle wasting in tumor-cachexia [10]. To this point, it should be mentioned, however, that there is also some caution about the use of peptidergic agents, and adaptation processes, particularly on a long-term basis [156]. There is some growth of several tumor cell lines (EGF) [157,158], and hyperplastic lesions in the colon (subjects treated with glucagon-like peptide 2) (GLP-2) [159]. Contrarily, BPC 157 shows evidence of well-controlled adaptive processes, with full recovery achieved in short bowel rats [18,119], strongly contrasting with inadequately controlled adaptive processes elsewhere [156,157,158,159]. Moreover, additional supportive evidence for BPC 157 (i.e., no toxic effect, limit test negative, lethal dose LD1 not achieved, no side-effects in trials (for review, see [1,3,4,5,6,8,9,32,33,34,35,36])) shows that it inhibits the growth of several tumor cell lines and counteracts the tumor-promoting effect of VEGF [142]. This strongly distinctive point is also appreciated in another review [160]. Thereby, there is an important demonstration that in mice with C26 colon adenocarcinoma, BPC 157 counteracted tumor-cachexia, muscle wasting, and markedly prolonged survival [10]. BPC 157 afforded significant mitigating action against cancer cachexia-induced muscle degeneration, inflammation, and catabolism. BPC 157 significantly corrected deranged muscle proliferation as well as myogenesis, counteracted an increase in proinflammatory and procachectic cytokines such as interleukin 6 (IL-6) and TNF-α implicated in muscle metabolism relevant to cancer cachexia, as well as any changes in the expression of FoxO3a, p-AKT, p-mTOR, and P-GSK-3β [10]. 

## 8. Conclusions

Taken together, the achieved healing and function recovery might approach the entire problem of muscular disturbance. The BPC 157–muscle relation, in particular its wide theoretical and practical background and application, might resolve a very complex issue. Illustratively, the recovered purposive movement through the therapy represents a complex issue that was adequately resolved by the therapy (i.e., the impulses might effectively pass from the motor cortex via the spinal cord to the appropriate muscles and that the movement pattern is effectively coordinated by the impulses passing through various parts of the brain and sending messages back to the motor cortex). 

**Figure 11:**
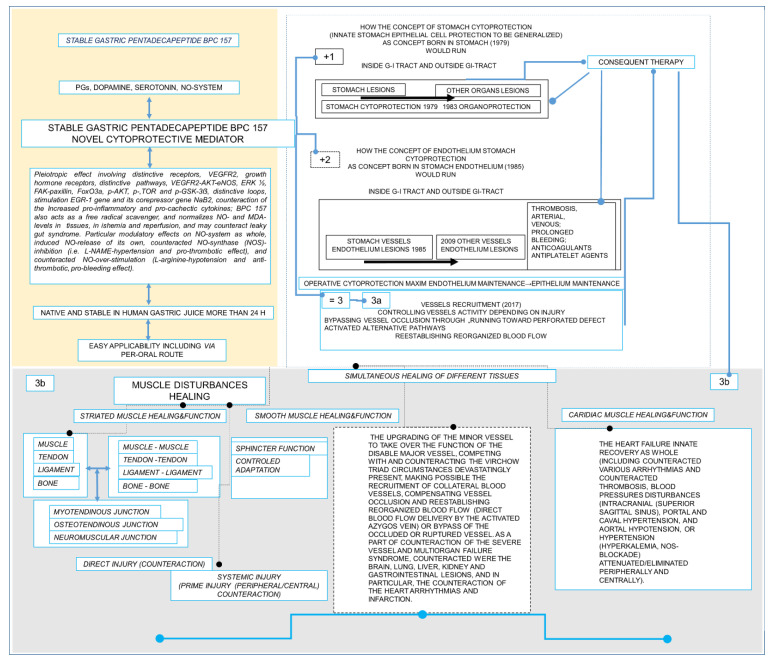
Summarization of particular way in which the stable gastric pentadecapeptide BPC 157 might beneficially act on striated, smooth, and heart muscle. *BPC 157 (yellow box).* Stable gastric pentadecapeptide BPC 157, a peptide that is native to and stable within human gastric juice for periods of time longer than 24 h, has demonstrated extremely positive healing effects for various injury types in numerous organ systems, and muscle systems in particular. Providing specific effects in conditions of sickness [58,96], and also at the molecular level, BPC 157 accelerated the ex vivo outgrowth of tendon explant in culture and increased the migratory potential of cultured tendon fibroblasts through the activation of an FAK-paxillin pathway. The growth hormone receptor occurred as one of the most abundantly up-regulated genes in tendon fibroblasts by BPC 157 through Janus kinase 2, the downstream signal pathway of growth hormone receptor [59]. BPC 157 enhanced the proliferation of human umbilical vein endothelial cells (HUVECs). BPC 157 regulated the phosphorylation level of extracellular signal-regulated kinases 1 and 2 (ERK1/2) as well as their downstream targets, including c-Fos, c-Jun, and early growth response gene-1 (egr-1), which are key molecules involved in cell growth, migration, and angiogenesis, respectively [62]. In another assay with non-differentiated Caco-2 cells, stimulation of the expression of egr-1 was, along with stimulation of its repressor nerve growth factor 1-A binding protein-2 (nab2), an effect exerted more rapidly than platelet-derived growth factor subunit B (PDGF-BB) [63]. In the counteraction of the hind limb ischemia, the accelerated blood flow recovery and vessel number in rats with hind limb ischemia [61] might be the result of the up-regulation of VEGFR2 expression in rats with hind limb ischemia and endothelial cell culture, as well as the promotion of VEGFR2 internalization in association with VEGFR2-AKT-eNOS activation [61]. Additionally, this might be control of the vasomotor tone and the activation of the Src-Caveolin-1-eNOS pathway [60,61]. As part of BPC 157’s counteraction of the procachectic and proinflammatory cytokines pathways, the counteraction of tumor-induced muscle cachexia and the signaling processes implicated in cancer cachexia [10] and leaky gut [11], as well as its role as a membrane stabilizer and free radical scavenger have been reported (for review see, [10,11]). As emphasized, its particular stability and safety, and thereby easy application, might make possible the realization of these particular molecular pathways in practical cytoprotection therapy (for review, see [1,3,4,5,6,8,9,32,33,34]). *Cytoprotection* and *BPC 157 (white box).* The cytoprotection concept (for review, see [35,36,37,38,39,40,41,42]), born in the stomach, appears with the application of cytoprotective agents as innate stomach epithelial cell protection (indicated as +1) to be generalized (Robert, Szabo) in other epithelia protection in other organs (organoprotection) supplemented by stomach endothelium cell protection (indicated as +2) (Szabo). Together (+1, +2), these result in the cytoprotection stomach principle ‘endothelial maintenance → epithelial maintenance’ (indicated as = 3) as an axis for a rapid defensive response to resolve ongoing lesions, which is, however, not fully operative with the standard cytoprotective agents. As a novel cytoprotection mediator, BPC 157 might exert prominent beneficial epithelial effects in the stomach and in the whole gastrointestinal tract as well as in other organs (stomach cytoprotection → organoprotection) (+1) and endothelial beneficial effects in the stomach (+2). Therefore, BPC 157 therapy might make the stomach principle ‘endothelial maintenance → epithelial maintenance’ (indicated as = 3) as a fully operative axis (indicated as 3a). Further, BPC 157 therapy might extend the original cytoprotective maxim to ‘endothelial maintenance → epithelial maintenance’ from the stomach to the endothelial protection of other vessels (3a) (for review, see [1,3,4,5,6,8,9,32,33,34]). In this, BPC 157 might induce particular vessel recruitment and activation depending on injury, i.e., when confronted with vessel occlusion, there was collateral activation to bypass vessel occlusion, and, when confronted with a perforation defect, vessels “running“ toward the defect (3a) (for review, see [1,3,4,5,6,8,9,32,33,34]). The rapid result is the re-establishment of the reorganized blood flow (3a). As a consequence, a particular therapy might beneficially affect thrombosis, both arterial and venous, and lesions presentation. *Cytoprotection concept extended to the muscular disturbances healing with BPC 157 (gray box) (3b).* Simultaneous healing occurred as a particular point of the realized cytoprotection healing course. Therefore, BPC 157 therapy might induce a particular upgrading of the minor vessel to take over the function of the disabled major vessel, resolving Virchow triad circumstances that may be devastatingly present. Consequently, this might make possible the collateral vessels activation, compensating the function of the major vessel and reestablishing reorganized blood flow (direct blood flow by the activated azygos vein) (indicated as box with dashed frame) (for review, see [1,3,4,5,6,8,9,32,33,34]). This might be crucial for the described muscle healing and function recovery of striatal and smooth muscle, as well as heart failure recovery with BPC 157 therapy (for review, see [1,3,4,5,6,8,9,32,33,34]). This therapy (3b) might be the realization of the multimodal muscle axis impact that is able to react depending on the condition and the given agent(s) and the symptoms that are distinctively related to the primary cause of injury within the wider healing concept, and the concept of cytoprotection in particular.

Such a conclusion about the theoretical BPC 157–muscle relation might illustrate a quite large range of the observable recovery with BPC 157 therapy. There was a consistent chain of events for the recovered muscle disabilities, all within a wide cytoprotection background, with well-defined therapeutic effects of BPC 157 application. This point is summarized in Figure 11.

*Stable gastric pentadecapeptide BPC 157 is a partial sequence of the human gastric juice protein BPC, which is freely soluble in water at pH 7.0 and in saline. BPC 157 (GEPPPGKPADDAGLV, molecular weight 1419; Diagen, Slovenia) was prepared as a peptide with 99% high-performance liquid chromatography (HPLC) purity, with 1-des-Gly peptide being the main impurity. PGs, prostaglandins; NO, nitric oxide; VEGF, vascular endothelial growth factor; VEGFR2, VEGF receptor 2; eNOS, endothelial nitric oxide synthase; FAK, focal adhesion kinase; FoxO3a, transcription factor; p-AKT, phospho-AKT; p-mTOR, phospho mammalian target of rapamycin; p-GSK-3β, phospho glycogen synthase kinase 3β; MDA, malondialdehyde; GI, gastrointestinal.*

In this interconnected chain of events, there was a specific recording of the multimodal muscle axis presentation with BPC 157 therapy (Table 1).

This multitude (Table 1) clearly demonstrates a multimodal muscle axis impact that is able to react depending on the condition and the given agent(s) and the symptoms that are distinctively related to the primary cause of injury within the wider healing concept, where the implementation of the concept of the cytoprotection [1,3,4,5,6,8,9,32,33,34] has appeared as novel point. Thus, for BPC 157 therapy, a well-functioning cytoprotection-loop (i.e., brain-periphery) might provide the translation necessary for the preserved muscle function to consistently occur (see *Chapter 1*, *Chapter 2*, and *Chapter 3*). This was further extended with the additional BPC 157–muscle relations via smooth muscle (see, *Chapter 4*) and cardiac muscle (see, *Chapter 5*) function recovery. Further support was given throughout the specific points, described with BPC 157 and cuprizone neurotoxin (mimicking multiple sclerosis-like disturbances in rats) (*Chapter 6*). Final emphasis regarding the full beneficial background was given with the BPC 157 and muscle wasting in tumor-cachexia (*Chapter 7*).

Furthermore, and importantly for muscle injury healing and function recovery, BPC 157 has an analgesic effect of its own [161,162,163,164]. It is of note that, consistently upon injury induction, the BPC 157-treated rats regularly exhibited fast function recovery (i.e., lack of leg contracture) (i.e., [43,44,45,46,47,48,93,94,95,96,97,98]). Thus, the noted analgesic effect might be part of the therapy’s rapid healing effect (i.e., lesser lesion, lesser pain). However, this effect might permit the animal to use the injured leg accordingly, and thereby, improve the healing as well. Illustratively, and likely as a part of its particular healing effect, BPC 157 produced analgesia in the MgSO_4_ and acetic acid test in mice, a model of prolonged pain associated with tissue injury [161], and counteracted succinylcholine-induced muscle pain (violent screaming upon light touch) in rats [102]. Indicatively for the possible translation (i.e., multiple types of knee pain), 11 of 12 patients had significant improvement in knee pain after one intra-articular injection of BPC 157 lasting more than one year thereafter [163]. However, this might antagonize morphine analgesia and haloperidol potentiation of the morphine analgesia [165]. In addition, and as mentioned, it might antagonize the effect of local anesthetics [103,104].

Thus, this review highlights a practical background that includes a peptide free of carrier. The stable gastric pentadecapeptide BPC 157 is a peptide that is always given alone, without any carrier, as a prototype of anti-ulcer cytoprotective peptide, native and stable in human gastric juice, used in clinical trial phase II (ulcerative colitis) and with a very safe profile (lethal dose LD1 not achieved) (for review, see [1,3,4,5,6,8,9,32,33,34]). The very safe profile might be taken as a definitive advantage, as recently confirmed in a large study conducted by Xu and collaborators [166]. Finally, by in situ hybridization and immunostaining, BPC 157 was found in human fetus and adult gastrointestinal mucosa, lung bronchial epithelium, epidermal layer of the skin and kidney glomeruli [1,9]. Thus, in addition to the previous suggestion that BPC 157 may have regulatory roles in the function of the human lung, kidney and skin [1,9], we could suggest a similar role of BPC 157 in muscle function regulation and therapy as well. Finally, pentadecapeptide BPC 157, as native and stable in human gastric juice, might be a prototype for an anti-ulcer cytoprotective peptide for muscle therapy. Given the high curing potential, very safe profile (lethal dose not achieved), and with a suitably wide effective range (µg-ng regimens) and unlimited methods of application (i.e., intraperitoneal, intragastric, in drinking water or topically, at the site of injury) (for review, see [1,3,4,5,6,8,9,32,33,34])), it may be a real challenge for further therapy.

Thus, to resolve the entire problem of muscle disturbances, we have presented the implementation of the cytoprotection theory and concept with the easy applicable BPC 157 as a prototype cytoprotective peptide model. We exemplified many targets—muscular, vascular and nerve, peripheral and central—that share functions with the multimodal muscle axis. However, these might be not a substitute for verification through large clinical trials. Nevertheless, in these wide, but instructive terms, several additional studies have recently emerged. It has been suggested that BPC 157, based primarily on animal model data, can act as a novel agent in the improvement of the clinical management of COVID-19 [167]. Pentadecapeptide BPC 157 efficiently reduces radiation-induced liver injury and lipid accumulation through Kruppel-like factor 4 upregulation both in vivo and in vitro [65]. Similar effects and roles have also been noted in other species (i.e., birds [168], and insects, such as honeybees [169,170]).

## Figures and Tables

**Table 1:** Multimodal muscle axis presentation with BPC 157 therapy confronted with various muscular disabilities. Note, BPC 157 therapy ranges within 10 µg and 10 ng/kg (GEPPPGKPADDAGLV, molecular weight 1419; Diagen, Slovenia).

Therapy Effect	Ref.
*Striated muscle*	
Recovered myotendinous junction	[43]
Recovered muscle injuries	[43,44,45,46,47,48]
Recovered tendon injuries	[48,58,59,94,95,96,97]
Recovered ligament injuries	[98]
Recovered bone injuries	[99,100,101]
Recovered vascular occlusion and occlusion-like disturbances	[66,71,72,73,74,75,76,77,78,79,80,81,82]
Recovered succinylcholine-induced disturbances	[102]
Recovered local anesthetic-induced disturbances	[103,104]
Recovered severe electrolyte-induced disturbances	[19,55,80,105,106]
Recovered disabled dopamine-, serotonin- and NO-system disturbances	[23,24,25,26,30,51,52,53,54,55,56,57]
Recovered spinal cord compression muscular disabilities	[28,29]
Recovered stroke muscular disabilities	[20]
Recovered traumatic brain injury muscular disabilities	[21]
Recovered reserpine muscular disabilities	[30]
Recovered MPTP (Parkinson-like) muscular disabilities	[30]
Recovered cuprizone (multiple sclerosis-like) muscular disabilities	[31]
Recovered tumor-induced cachexia	[10]
*Smooth muscle*	
Recovered function of disabled sphincters	[55,73,91,107,108,109,110,111,112,113,114,115,116,117,118]
Maintained normal sphincters’ function	[107,116,117]
Controlled adaptation, recovery of the short bowel rats	[18,119]
*Heart*	
*Recovery from heart failure as whole*	[66,71,72,73,74,75,76,77,78,79,80,81,82]
This might include the various counteracted arrhythmias and thromboses, or even blood pressures disturbances (intracranial (superior sagittal sinus), portal and caval hypertension, and aortal hypotension), or hypertension (hyperkalemia, NOS-blockade, attenuated/eliminated peripherally and centrally).	[66,67,68,69,70,71,72,73,74,75,76,77,78,80,81,82,102,103,105,106,122,123,154]

## Data Availability

The data presented in this study are available on request from the corresponding author.
